# SMAD1 as a biomarker and potential therapeutic target in drug-resistant multiple myeloma

**DOI:** 10.1186/s40364-021-00296-7

**Published:** 2021-06-16

**Authors:** Jian Wu, Min Zhang, Omar Faruq, Eldad Zacksenhaus, Wenming Chen, Aijun Liu, Hong Chang

**Affiliations:** 1 Department of Laboratory Medicine and Pathobiology, University of Toronto, Toronto, ON Canada; 2 Department of Hematology, Beijing Chaoyang Hospital, Capital Medical University, Beijing, China; 3 Department of Laboratory Hematology, Laboratory Medicine Program, University Health Network, Toronto, ON Canada

## Abstract

**Background:**

SMAD1, a central mediator in TGF-β signaling, is involved in a broad range of biological activities including cell growth, apoptosis, development and immune response, and is implicated in diverse type of malignancies. Whether SMAD1 plays an important role in multiple myeloma (MM) pathogenesis and can serve as a therapeutic target are largely unknown.

**Methods:**

Myeloma cell lines and primary MM samples were used. Cell culture, cytotoxicity and apoptosis assay, siRNA transfection, Western blot, RT-PCR, Soft-agar colony formation, and migration assay, Chromatin immunoprecipitation (Chip), animal xenograft model studies and statistical analysis were applied in this study.

**Results:**

We demonstrate that SMAD1 is highly expressed in myeloma cells of MM patients with advanced stages or relapsed disease, and is associated with significantly shorter progression-free and overall survivals. Mechanistically, we show that SMAD1 is required for TGFβ-mediated proliferation in MM via an ID1/p21/p27 pathway. TGF-β also enhanced TNFα-Induced protein 8 (TNFAIP8) expression and inhibited apoptosis through SMAD1-mediated induction of NF-κB1. Accordingly, depletion of SMAD1 led to downregulation of NF-κB1 and TNFAIP8, resulting in caspase-8-induced apoptosis. In turn, inhibition of NF-κB1 suppressed SMAD1 and ID1 expression uncovering an autoregulatory loop. Dorsomorphin (DM), a SMAD1 inhibitor, exerted a dose-dependent cytotoxic effect on drug-resistant MM cells with minimal cytotoxicity to normal hematopoietic cells, and further synergized with the proteasomal-inhibitor bortezomib to effectively kill drug-resistant MM cells in vitro and in a myeloma xenograft model.

**Conclusions:**

This study identifies SMAD1 regulation of NF-κB1/TNFAIP8 and ID1-p21/p27 as critical axes of MM drug resistance and provides a potentially new therapeutic strategy to treat drug resistance MM through targeted inhibition of SMAD1.

**Supplementary Information:**

The online version contains supplementary material available at 10.1186/s40364-021-00296-7.

## Introduction

Multiple myeloma (MM) is a hematologic neoplasm characterized by the clonal proliferation of malignant plasma cells. The clinical outcome of MM patients has remarkably improved in recent years, largely due to the introduction of the combination of bortezomib (BTZ) and Dexamethasone (DEX) in the frontline or relapsed/refractory clinical setting. However, relapse and drug-resistance often occur in MM patients. There is therefore an urgent and unmet demand to elucidate the molecular mechanisms of drug resistance and promote therapeutic efficacy of available drugs [1, 2].

Small Body Size (SMA) and Mothers Against Decapentaplegic family 1(SMAD1), also known as JV4–1/MADH1/MADR1, which maps to human chromosome 5q4 [3], was first identified in a study of genes involved in the pathogenesis of breast cancer [4], and later considered as an important regulator in tumor progression including MM [5]. SMAD1 mediates the signals of bone morphogenetic proteins (BMPs) [6], which are involved in a range of biological activities including cell growth, apoptosis, development and immune responses. BMP ligands induce SMAD1 phosphorylation and activation through the BMP receptor kinase. Phosphorylated SMAD1 forms a complex with SMAD4, which then translocates to the nucleus where it regulates gene transcription in cooperation with transcriptional factors [7].

Several studies highlight an important role for SMAD1 in promoting cell invasion and metastasis in different types of cancers [8]. For example, overexpression of SMAD1 induces proliferation in stomach cancer cells in response to BMP-7 stimulation [9], and upon activation by BMP-9, SMAD1 promotes ovarian cancer cell growth [10].

In MM, tumor cells are confined to the bone marrow microenvironment, and are exposed to high levels of TGF-β secreted from both MM cells and bone marrow stromal cells, leading to further activation of cell growth and survival pathways [11, 12]. Notably, it has been demonstrated that upregulation of SMAD1 and ID1 downstream of TGF-β signaling is indicative of activation of this pathway [13]. However, the clinical relevance of SMAD1 induction in MM and its role in drug resistance has not been reported. Dorsomorphin (DM), a specific SMAD1 inhibitor, prevents SMAD1 phosphorylation, leading to suppression of lung cancer cell growth [14, 15]. The cytotoxic effect of DM on MM, particularly in drug-resistant MM, has not been unexplored.

Here, we provide clinical and experimental evidence that highlights an important role of SMAD1 in MM through inhibition of p21 and p27 via ID1, and its effect on drug resistance. We also demonstrate a cross talk between SMAD1 and NF-κB1 through TNFAIP8. Moreover, we showed that DM blocks SMAD1 phosphorylation(p-SMAD1), leading to diminished survival of MM cells, and that addition of DM to standard of MM therapy (BTZ) effectively kill drug-resistant MM in a pre-clinical model.

## Material and methods

### Myeloma cell lines and primary MM samples

The MM parental cell lines (RPMI-8226, MM1.S), and MM1.R (DEX resistant), OPM2 vel/R (BTZ resistant), were obtained from ATCC. RPMI-8226R5, a multidrug-resistant MM cell line with cross-resistance to BTZ, was kindly provided by Dr. R Buzzeo [16]. The resistance of both RPMI-8226R5 and MM1.R to the proteasome inhibitors BTZ was demonstrated in our previous study [17]. All cell lines were cultured in complete RPMI-1640 medium supplemented with 10% FBS as described previously [18]. Primary mononuclear cells were freshly isolated and purified from the bone marrow of MM patients, with the institution research ethic board approval.

### Cell culture, cytotoxicity and apoptosis assay, siRNA transfection, Western blot, RT-PCR, Soft-agar colony formation, and migration assay, Chromatin immunoprecipitation (Chip), animal xenograft model studies and statistical analysis

These assays and analyses are detailed in supplementary methods.

## Results

### SMAD1 is elevated in the malignant cells of a subgroup of MM patients and its overexpression predicts disease progression and poor survival

**Fig. 1:**
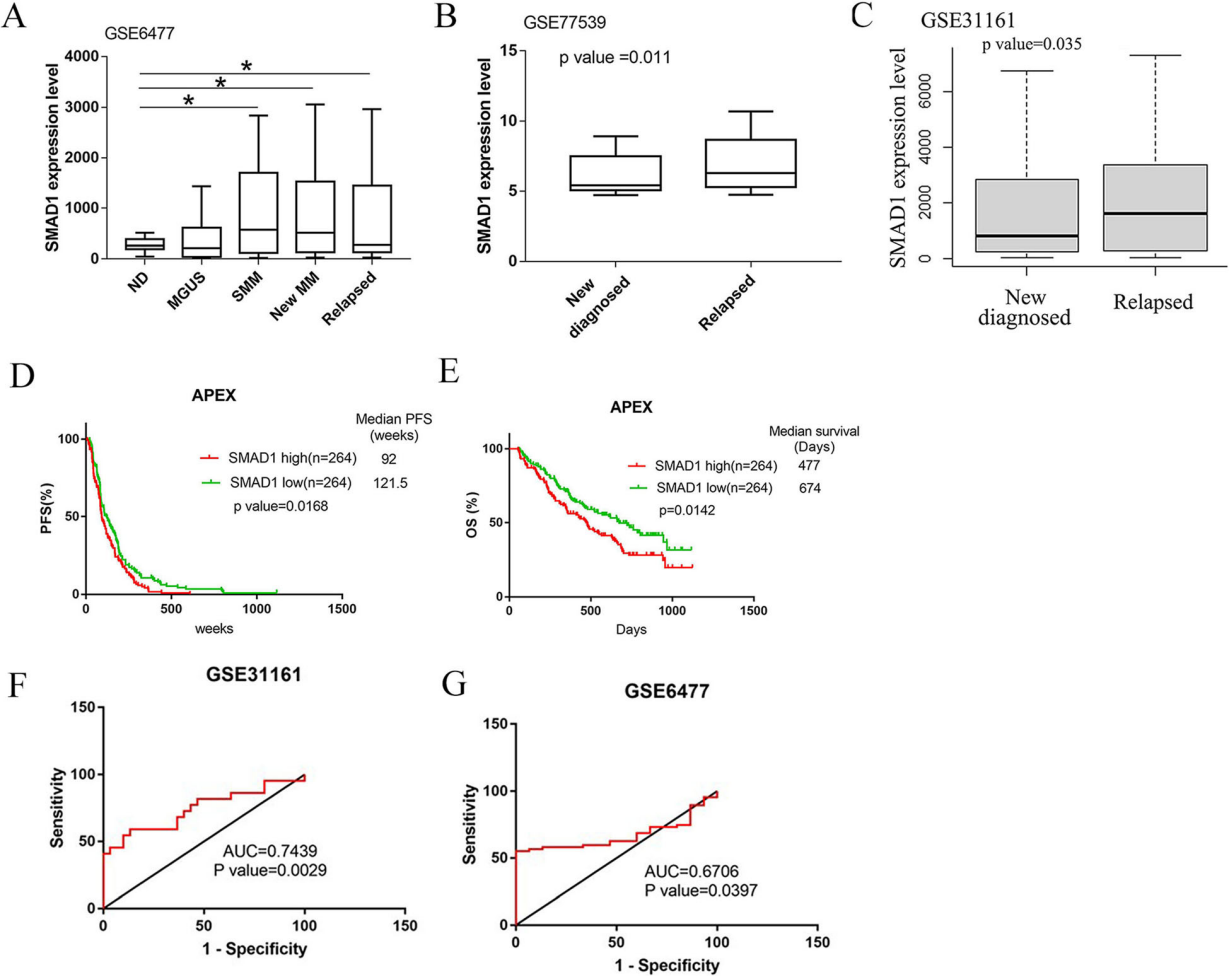
SMAD1 is elevated in a subpopulation of MM patients and its overexpression correlates with disease progression and poor survival. **a** SMAD1 expression in different stages of MM as shown in graph, GSE6477 dataset (ND *n* = 15, MGUS *n* = 22, SMM *n* = 24, newly diagnosis MM *n* = 73 and Relapse MM *n* = 28) **b** SMAD1 expression in primary MM cells from GSE77539 data set(*n* = 34 pair sample from 17 MM patients in diagnosis and relapse). One-way analysis of variance and paired sample T test was used, *p* < 0.05 is significant. **c** The box whisker plot shows SMAD1 expression in primary multiple myeloma plasma cells from patients treated by total therapy 2, 3 and other protocols at baseline and relapse, GSE31161 dataset (Sample *n* = 1038, 781 baseline and 256 relapse MM patients). **d, e** Kaplan-Meier plots indicate the overall survival (OS)(D) and progression free survival (PFS)(F) for 528 MM patients in APEX clinical trials (GSE9782) categorized by SMAD1 expression level(*n* = 264 for low-SMAD1 group versus *n* = 264 for high-SMAD1 group), *p* value is determined by log-rank test. **f** ROC curve for the SMAD1 to separate new diagnosis from relapse MM patients. **g** ROC curve for the SMAD1 to separate new diagnosis MM patients from healthy donors

Given the evidence associating high SMAD1 expression with oncogenesis in other types of cancers, we investigated its effect in MM patients. Using Gene Expression Omnibus (GEO) datasets, we observed a significant increase in the expression of SMAD1 in advanced stages of MM (GSE6477, relapse MM vs normal donor (ND) *p* = 0.047; newly diagnosed MM vs ND *p* = 0.032) (Fig. 1a). We also analyzed data of 17 paired samples from MM patients at diagnosis and relapse and found that SMAD1 was overexpressed in relapsed MM (GSE77539, *p* = 0.011) (Fig. 1b). These findings were further confirmed in an independent GSE dataset (GSE31161, *p* = 0.0064) (Fig. 1c).

To investigate the prognostic significance of SMAD1 overexpression in MM development and progression, we evaluated SMAD1 gene expression based on the APEX trial GEO microarray database (GSE9782). High SMAD1 expression correlated with shorter median progression-free survival (PFS) and overall survival (OS) (PFS: 92 vs 121.5 weeks, *p* = 0.0168; OS: 477 vs 674 days, *p* = 0.0142) (Fig. 1d and e).

To examine the potential of SMAD1 as a biomarker for relapsed MM patients, we performed a receiver operating characteristic (ROC) analysis on GSE31161 (MM patients treated by Total Therapy 2, 3 and other protocols at baseline and relapse). The ROC curves of SMAD1 revealed strong significant discrimination between relapse MM and newly diagnosed MM, with AUC (area under the curve) of 0.7439 (Fig. 1f). Additionally, SMAD1 expression distinguished between newly diagnosed MM and normal bone marrow donors in the GSE6477 dataset, with AUC of 0.6706 (Fig. 1g). These results further establish SMAD1 as a valuable biomarker for MM patients across the course of the disease.

To determine whether SMAD1 is involved in regulating MM proliferation and migration, we suppressed p-SMAD1 expression using DM. As expected, downregulation of SMAD1 expression led to reduced colony formation in soft agar of 8226R5 and OPM2 vel/R cells (Fig. S1A). In addition, downregulated SMAD1 expression reduced migration in transwell assays. (Fig. S1B). Addition of TGF-β reversed the effect of siRNA-mediated SMAD1 depletion on migratory capacity of 8226R5 and OPM2 vel/R cell lines (Fig. S1C). These data support the notion that SMAD1 promotes the proliferation and migration of MM cells.

### BTZ and DEX responder express low levels of SMAD1 and other KEGG apoptosis related genes

**Fig. 2:**
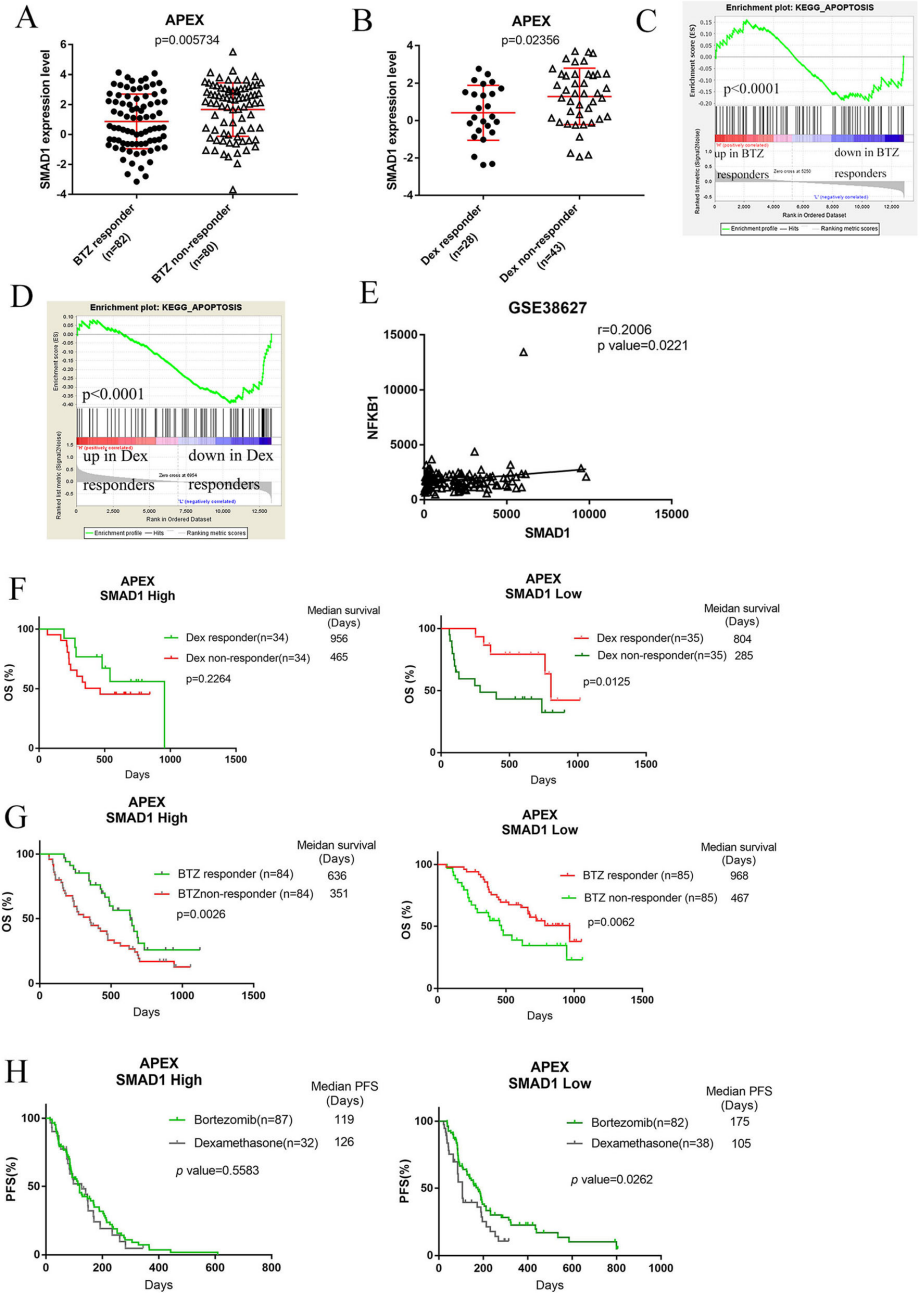
Lower SMAD1 expression is associated with improved OS following dexamethasone or bortezomib therapy. **a, b** Gene expression plots of SMAD1 (probe set 210993_s_at) in responders and non-responders based on Dex (*n* = 71) and BTZ (*n* = 162) treatment in the APEX trial. The red horizontal lines indicate the average value in the group and the standard deviation. *p*-value was calculated using a 2-tailed Mann-Whitney test. **c, d** GSEA plot supporting the downregulation of the genes in the KEGG pathway apoptosis in Dex and BTZ responders. **e** Correlation analysis of SMAD1 with NF-ΚB1expression in the patient dataset (GSE38627) presented as scatter plots. **f** Kaplan-Meier curves comparing OS of SMAD1 high (left) and low (right) expressing patients for Dex responder vs Dex non-responder arms in the APEX trial. **g** Comparing OS of SMAD1 high (left) and low (right) expression patients for BTZ responder vs BTZ non-responder arms in APEX trial. **h** Comparing PFS of SMAD1 high (left) and low (right) expressing patients for BTZ versus Dex arms in the APEX trial. Low and high expression is defined here as below and above the median. All *p* values were calculated with the Log-rank test

We next investigated whether SMAD1 expression is associated with response to standard MM treatment, containing BTZ and Dex [19]. In the APEX clinical trial, relapsed patients were treated with BTZ or Dex as a single agent. In the BTZ and Dex arm of the trial, SMAD1 mRNA expression was significantly lower in responders than in non-responders (Fig. 2a and b). The top pathway enriched by Gene set enrichment analysis (GSEA) in APEX trial dataset by comparing the SMAD1-high and SMAD1-low expression samples was ‘Apoptosis’ pathway. The other pathway were KEGG_ENDOMETRIAL_CANCER,KEGG_INTESTINAL_IMMUNE_NETWORK_FOR_IGA_PRODUCTION,KEGG_VIRAL_MYOCARDITIS,KEGG_CHRONIC_MYELOID_LEUKEMIA(Fig. 2c and STable1). GSEA of the Dex branch of the trial also revealed a significant enrichment of the KEGG Apoptosis pathway (Fig. 2d). Correlation analysis was performed on individual components of the KEGG Apoptosis pathway including Casp3, Casp6, NF-κB1. Only NF-κB1 expression positively correlated with SMAD1 expression (Fig. 2e), suggesting that SMAD1 expression is not only a biomarker for treatment response, but also is potentially a regulator of drug sensitivity in MM patients through regulation of NF-κB1 (see below).

### Low SMAD1 expression is associated with longer survival of MM patients receiving BTZ and/or Dex treatment

The association between SMAD1 RNA expression levels and clinical BTZ and Dex response raised the question of whether SMAD1 levels are associated with the survival benefit of BTZ and Dex treatment. To address this question, data from APEX clinical trial were analyzed. In this trial, relapsed patients were treated with BTZ or Dex as single agents. We divided the patients from the APEX trial into two groups based on SMAD1 expression levels, either below or above the median, and then compared survival in Dex-responder versus non-Dex responder. OS of patients with high SMAD1 expression was not statistically influenced when they were treated on the Dex arm (956 days vs 456 days, *p* = 0.2264) (Fig. 2f left). In contrast, the OS of patients with low SMAD1 expression was significantly longer in the Dex-responsive arm (804 days vs 285 days, *p* = 0.0125) (Fig. 2f right). Similar effects on OS (636 vs 351 days) but not PFS (median 119 vs 126 days, *p* = 0.5583) were observed in patients treated with BTZ (Fig. 2g, h).

### The relationship between SMAD1 and apoptosis pathway in MM

To examine if there is a potential correlation between SMAD1 expression and activation of the apoptotic pathway in MM, we analyzed the Cancer Cell Line Encyclopedia (CCLE), a large database of gene expression profiling for more than 1000 human cancer cell lines. We generated a Z score for each cell line through KEGG Apoptosis pathway gene sets. We found a moderate but highly significant negative correlation between high SMAD1 expression and KEGG Apoptosis pathway inhibition across the ~ 1000 CCLE cell lines (*R* = -0.1486, *p* < 0.0001) (Fig. S2A); an enrichment of apoptosis inhibition (high Z score) was also observed in other hematological malignancies (Fig. S2 B-F). Taken together, these results establish a strong negative correlation between SMAD1 RNA expression and the apoptotic pathway activity in MM.

### SMAD1 is required for MM cell growth and confers drug resistance upon MM cells

**Fig. 3:**
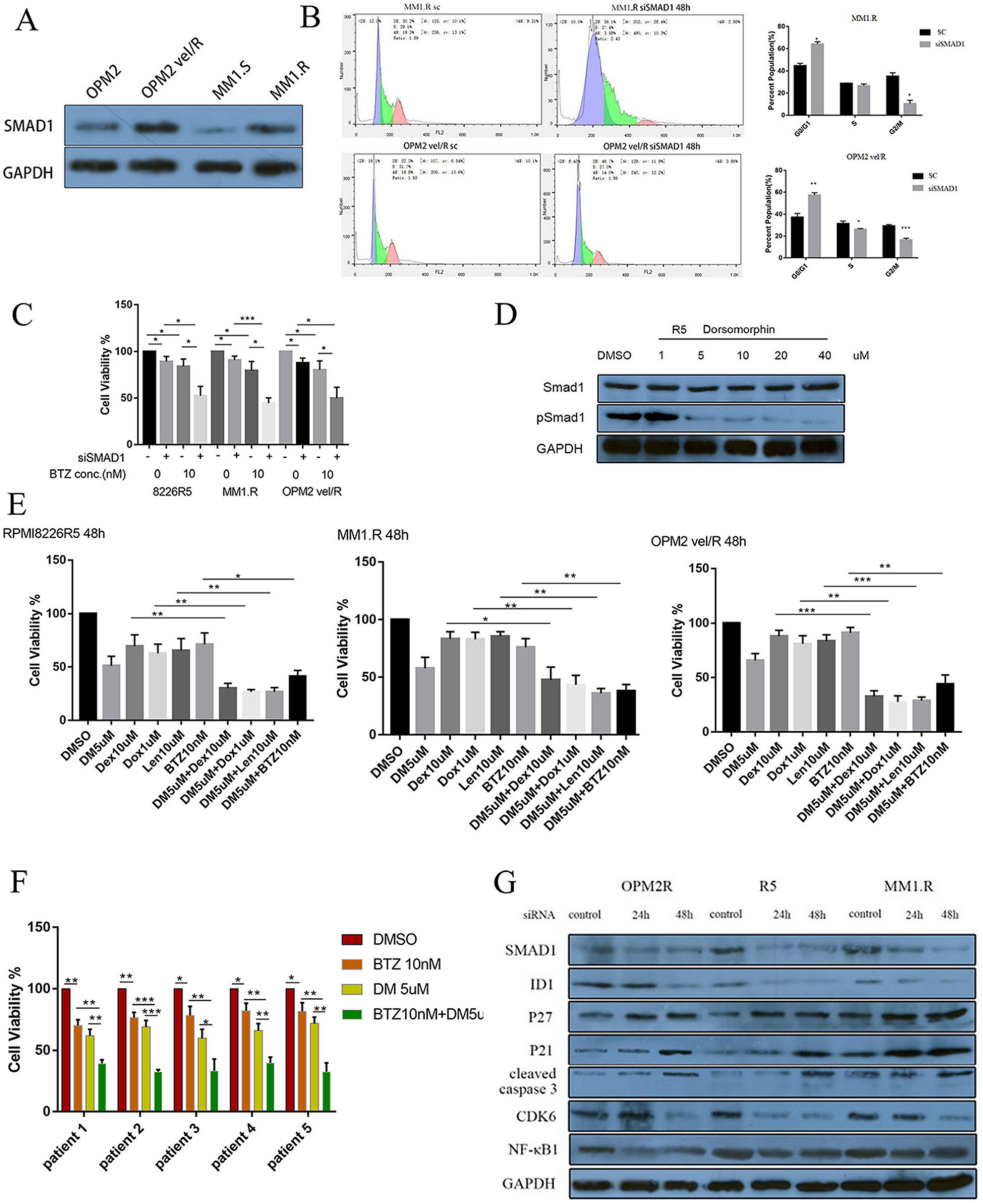
SMAD1 is required for MM cell growth and its over-expression confers drug resistance upon MM cells. **a** SMAD1 expression was determined by western blot analysis in MM1.R and OPM2 vel/R. (**b** left) Representative results showing SMAD1 knockdown induces cell cycle arrest in MM1.R and OPM2 vel/R. MM1.R and OPM2 vel/R were transfected with si-SMAD1 30 nM or siRNA control for 48 h, then the cells were subjected to cell cycle analysis. (**b** right) Quantitative results of the cell cycle phase of results. **c** The 8226 R5, MM1.R and OPM2 vel/R cell lines were transfected with synthetic si-SMAD1 50 nM or siRNA control and treated with different concentrations of BTZ for 48 h and cell viability was measured using MTT assay. **d** 8226 R5 was treated with indicated different concentration of DM for 24 h and SMAD1 phosphorylation was assessed by western blot. **e** 8226R5, MM1.R and OPM2 vel/R cells were treated with indicated concentrations of DM and anti-myeloma drug alone, or combination for 48 h and cell viability was assessed by MTT assay. **f** Combination of drugs (DM, BTZ) synergistically induces cytotoxic effects on primary mononuclear cells MM patients’ samples. Primary mononuclear cells derived from 5 MM patients were treated with indicated concentration of BTZ and DM for 48 h, and then cell viability was evaluated by MTT assays. Results are presented as means±SD. from at least three separate experiments. *: *p* < 0.05; **: *p* < 0.01; ***: *p* < 0.001. **g** CD138 + cells derived from 2 MM patients were treated with indicated concentration of BTZ, and DM for 48 h, and then cell viabiility was evaluated by MTT assays. Results are presented as means±SD. from at least three separate experiments. *: *p* < 0.05; **: *p* < 0.01; ***: *p* < 0.001. **h** 8226R5, MM1.R and OPM2 vel/R were transfected with si-SMAD1 50 nM or siRNA control. Protein lysate was subjected to western blot with indicated antibodies

To identify potential mechanisms by which SMAD1 might confer drug resistance in MM, we first determined endogenous SMAD1 expression in two drug-resistant MM cell lines (MM1.R, OPM2 vel/R) and their ancestors (MM1.S, OPM2). Immunoblotting showed enhanced SMAD1 protein expression in drug-resistant MM cell lines (MM1.R, OPM2 vel/R) compared to their parental drug-sensitive cells (MM1.S, OPM2) (Fig. 3a). Knocking down SMAD1 with siRNA in MM cell lines induced cell cycle arrest at the G0/G1 phase of the cell cycle (Fig. 3b). Western blot was performed in parallel to measure the efficiency of siRNA (Fig. S7A). SMAD1 silencing also enhanced the sensitivity of resistant MM cells to anti-myeloma drug BTZ (Fig. 3c). In addition, selective SMAD1 inhibition via DM decreased the presence of p-SMAD1 in drug-resistant cells (Fig. 3d). The combination treatment of DM plus the anti-myeloma drugs, BTZ (10 nM), Dex (10uM), lenalidomide (Len) (10uM), or doxorubicin (Dox) (1uM), resulted in synergistic death in both of the drug resistant MM cell lines (Fig. 3e and Fig. S3).

Cytotoxicity of anti-myeloma drugs in combination with DM was further investigated on primary MM cells derived from MM patients. Mononuclear cells and CD138 + cells from MM patients were treated for 48 h by BTZ, Dex, Len, Dox, alone and in combination with DM (Fig. 3f & g and Fig. S4 A & B). Co-treatment with anti-myeloma drugs and DM killed primary MM cells more effectively than either drug alone. To evaluate off-target cytotoxicity of DM treatment alone or together with BTZ on normal blood cells, PBMCs collected from three healthy donors were treat with DM or the combinations of DM plus BTZ 48 h. Cell viability of healthy PBMCs was not significantly compromised by DM concentration that are effective against MM. DM plus BTZ combinations showed some reduced cell viability comparing to the negative control group, but not compared to the BTZ treated arm (Fig. S4C). These results, along with those acquired in MM cell lines, revealed that depletion of p-SMAD1 renders MM cells more vulnerable to anti-myeloma drugs.

To determine the underlying molecular mechanisms of SMAD1-mediated myelomagenesis, we examined the protein level of SMAD1 in RPMI8226R5, MM1.R, and OPM2 vel/R cells transiently infected with si-SMAD1(Fig. 3h). Previous studies demonstrated that cell cycle-dependent kinase inhibitors p21^Cip1^ and p27^Waf1^ are transcriptionally suppressed by ID1, a downstream gene of SMAD1 [20, 21]. Indeed, ID1 protein decreased, whereas expressions of p21^Cip1^ and p27^Waf1^ were induced following depletion of SMAD1. Altogether, our results indicated that high SMAD1 expression in MM cells sustains proliferation and inhibits apoptosis through activation of the ID1/p21/p27 pathway.

### TGF-β enhances ID1/TNFAIP8 expression and suppresses apoptosis through a crosstalk between SMAD1 and NF-κB1

Given our aforementioned finding of positive correlation of SMAD1 and NF-κB1, the Chip-seq peaks which target SMAD1 in K562 and GM12878 cell lines were visualized using UCSC genome browser (http://genome.ucsc.edu) based on Encyclopedia of DNA Elements (ENCODE) data. Analysis of the NF-kB1 promoter region for consensus binding sites revealed direct recruitment of SMAD1 and overlap with H3K27Ac, an epigenetic mark associated with transcriptional activation (Fig. S5A). Indeed, 16 putative SMAD1 binding sites were identified in the region of the proximal region of NF-κB1 (Fig. S5B & C).

**Fig. 4:**
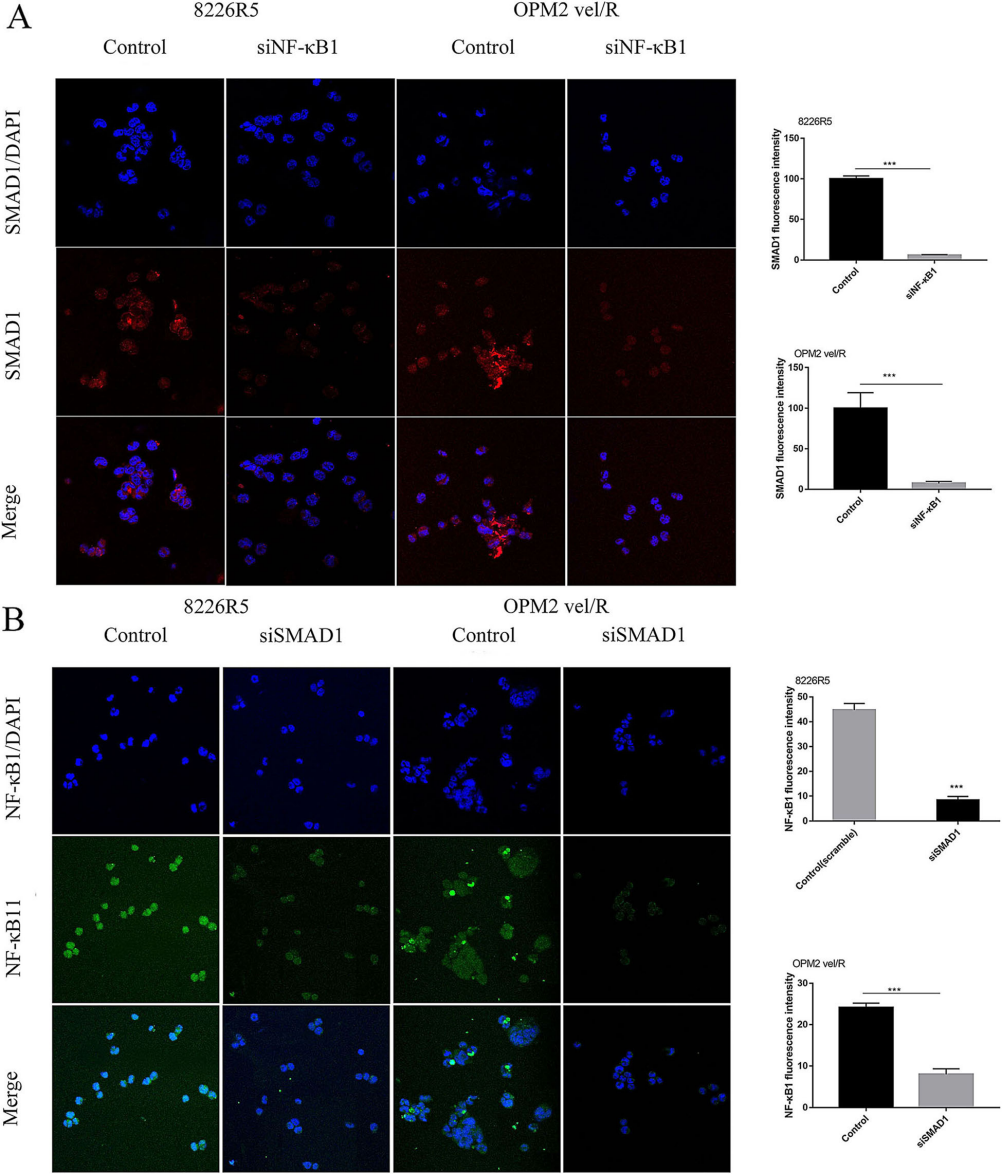
Interaction and cross regulation between SMAD1 and NF-κB1 in MM cells. **a** Representative images of SMAD1 in MM cells treated with control or 50 nM siNF-κB1. Bar graph display the results of the mean intensity of SMAD1 immunofluorescence of two groups. **b** Representative images of NF-κB1 in MM cells treated with control or 50 nM siSMAD1. Bar graph display the results of the mean intensity of NF-κB1 immunofluorescence of two groups

To determine whether SMAD1 could regulate NF-κB1, MM cells were transfected with either SMAD1 or NF-κB1 siRNA. When SMAD1 was depleted, NF-κB1 expression was also downregulated, and vice versa, suggesting a cross talk between these two transcription factors (Fig. 3g). In support of our Western blot results suggesting SMAD1 and NF-κB1 regulate each other’s expression, immunofluorescence staining demonstrated reduced SMAD1 protein following NF-κB1 knockdown in 8226R5 and OPM2 vel/R cell lines, and vice versa (Fig. 4). Western blot was performed in parallel with immunofluorescence to detect the expression of SMAD1 and NF-κB1(Fig. S7B).

**Fig. 5:**
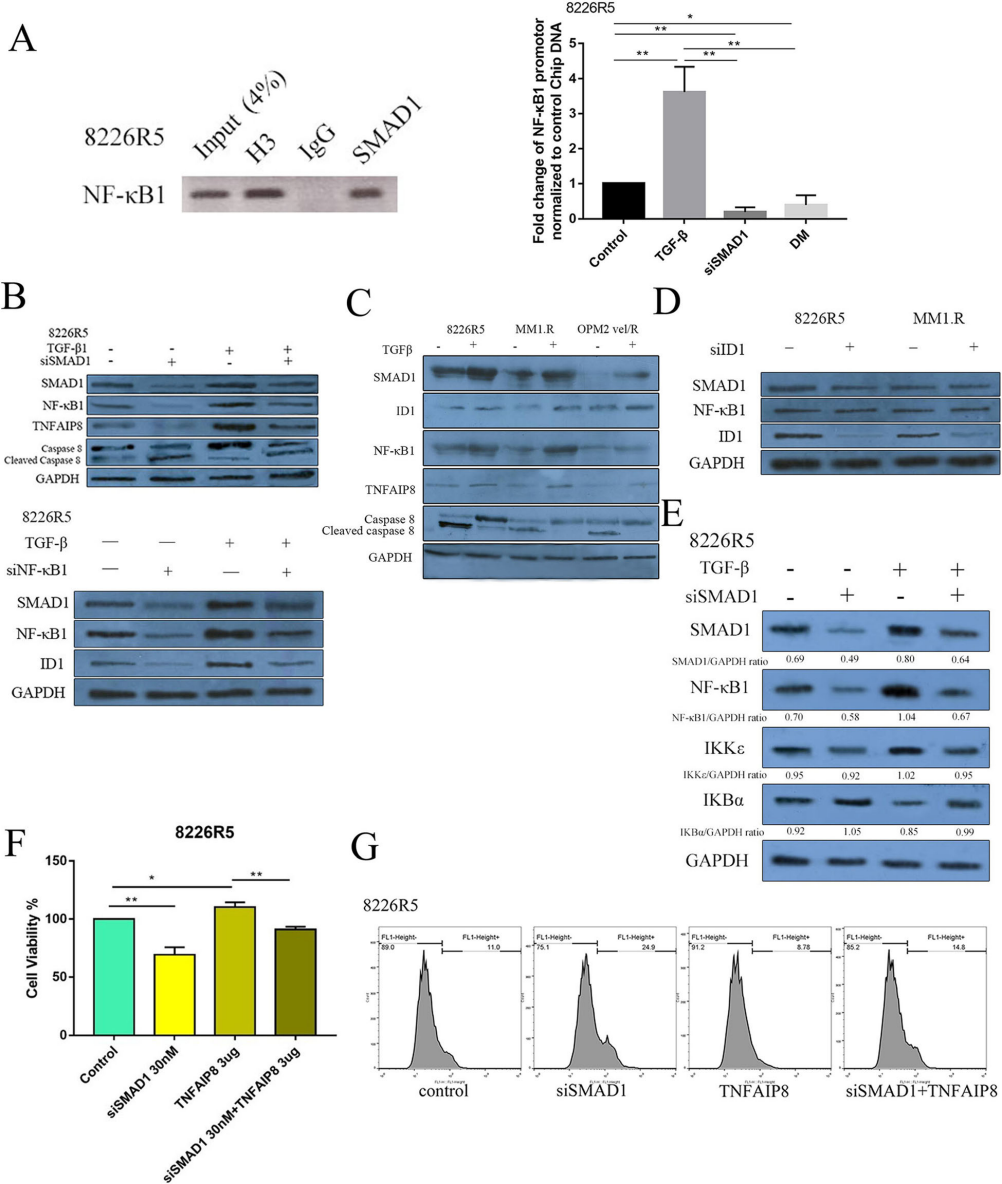
TGF-β enhances ID1/TNFAIP8 expression and inhibits apoptosis through a crosstalk between SMAD1 and NF-κB1. **a** A ChIP assay demonstrating SMAD1 antibody immunoprecipitates the NF-κB1 promoter in 8226R5 cells. H3 and igG antibodies were used as positive and negative control, respectively. **b** 8226-R5 cells were transfected with si-NF-κB1, si-SMAD1 or siRNA control (48 h), with or without TGF-β stimulation (6 h). The cell lysate was prepared and subjected to western blot with indicated antibodies. **c** The western blot analysis represents the indicated protein levels in RPMI-8226R5, MM1.R and OPM2 vel/R stimulated with TGF-β. **d** 8226-R5 and MM1.R cells were transfected with si-ID1 or siRNA control. The cell lysate was prepared 48 h after transfection and subjected to western blot with indicated antibodies. **e** Down-expression of SMAD1 alleviates TGF-β induced IKKε activation and IKBα degradation in 8226R5 cells. 8226R5 cells were transfected with si-NF-κB1, si-SMAD1 or siRNA control (48 h), with or without TGF-β stimulation (6 h). The cell lysate was prepared and subjected to western blot with indicated antibodies. **f** Cells were transiently co-transfected with reporter plasmids (pcDNA3.1 vector or TNFAIP8 plasmid) with siSMAD1 using liposome-3000 transfection reagent for 24 h. and then cell viability was evaluated by MTT assays. Results are presented as means±s.d. from at least three separate experiments. *: *p* < 0.05; **: *p* < 0.01; ***: *p* < 0.001; NS: not significant. **g** The cells were transiently co-transfected with reporter plasmids (pcDNA3.1 vector or TNFAIP8 plasmid) with siSMAD1 using liposome 3000 transfection reagent for 24 h, and then stained with annexin-V/propidium iodide and analyzed by flow cytometry to determine the percentage of apoptotic cells

This cross-regulation was confirmed by chromatin immunoprecipitation (ChIP) showing that SMAD1 binds to the promoter of NF-κB1 in 8226R5 cells (Fig. 5a, left). Treatment of 8226R5 cells with 2 ng/ml TGF-β significantly increased the amount of NF-κB1 promoter DNA that was ChIPed by SMAD1 antibody (Fig. 5a, right). There was also a significant decrease of the NF-κB1 promoter band in 8226R5 treated with 50 nM siSMAD1 or 10uM DM relative to control (Fig. 5a, right). These findings indicate that the NF-κB1 promoter is directly regulated by SMAD1 upon TGF-β stimulation.

Next, we carried out a rescue experiment where we induced SMAD1 and NF-κB1 expression by TGF-β after knockdown of SMAD1 or NF-κB1. These rescue experiments showed that TGF-β induced SMAD1 and NF-κB1 expression leading to induction of TNFAIP8 and ID1. Furthermore, this rescue led to reduction in the level of cleaved caspase 8, a marker for extrinsic apoptosis (Fig. 5b). We observed that SMAD1 and NF-κB1 protein levels were elevated in MM cells stimulated by TGF-β.

In addition to SMAD1, expression of its target gene, ID1, was increased, whereas expression of the CDK-inhibitors p21 and p27 levels were reduced. These results are consistent with previous studies showed that TNFAIP8 is a downstream mediator of NF-κB1-induced oncogenesis [22], and our showing that downregulation of TNFAIP8 could induce apoptosis by increasing caspase 8 levels in MM cells [23].

To determine the downstream consequences of increased NF-κB1 by TGF-β stimulation, we evaluated the levels of TNFAIP8 and activated caspase 8. Both TNFAIP8 and ID1 were elevated, while cleaved caspase 8 was reduced (Fig. 5c). Notably, siRNA mediated knockdown of ID1 did not affect levels of SMAD1 or NF-κB1(Fig. 5d), suggesting ID1 is downstream of these factors.

To explore the relationship between SMAD1 and NF-κB1, we examined whether SMAD1 affects NF-κB signaling. We found that TGF-β increased the level of IKKε and decreased expression of IκBα. In addition, knockdown of SMAD1 rescued the effect of TGF-β induced IKKε activation, and IκBα degradation in 8226R5 cells (Fig. 5e). These results suggest that increased SMAD1 expression regulates NF-κB1 through the induction of IKKε, upstream of NF-κB activation, and the inhibition of IκBα.

To further examine whether TNFAIP8 can increase cell viability and rescue cytotoxicity induced by SMAD1 depletion, we co-transfected MM cells with si-SMAD1 and TNFAIP8-expressing plasmid. Over-expression of TNFAIP8 rescued the effect of siSMAD1 on cell viability, further demonstrating its role downstream of the TGF-β-SMAD1 axis (Fig. 5f, g). In aggregate, these findings indicate that TGF-β enhanced survival through crosstalk between SMAD1 and NF-κB1 and regulation of TNFAIP8 and ID1 expression.

### DM suppresses tumorigenesis in a preclinical model of MM

**Fig. 6:**
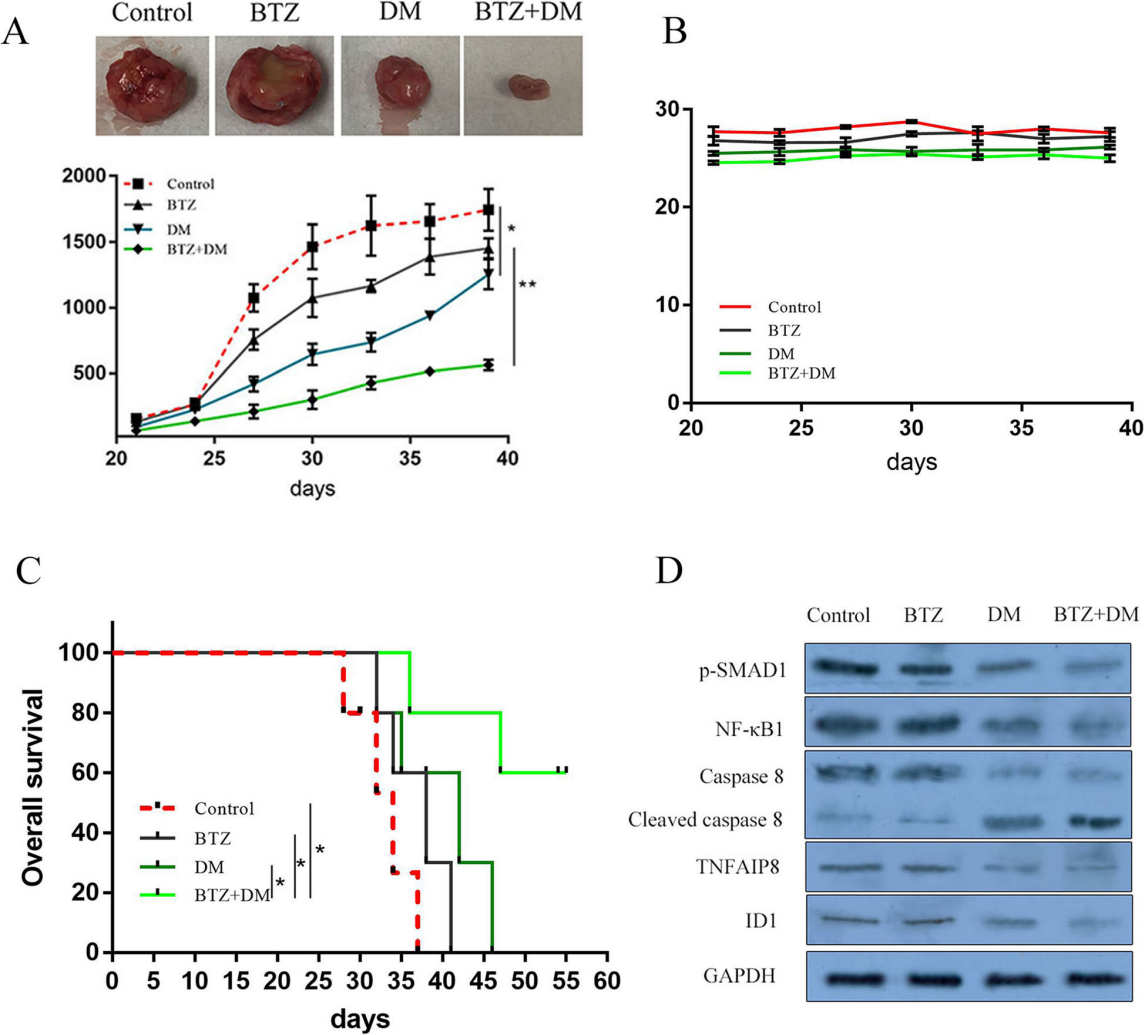
DM attenuates tumor growth and prolongs survival of mice injected with drug-resistant human MM cells. **a** DM enhanced BTZ-induced attenuations of tumor growth in severe combined immunodeficient mice. Representative images of immune-compromised mice with subcutaneous MM tumor. **b** Body weight was measured every 3 days and presented as means ± SD. **c** Overall Survival was evaluated using Kaplan-Meier curve and long-rank analysis from the first day of tumor cell injection until death or occurrence of an event. **d** Tumors treated as above were analyzed by immunoblotting with indicated antibodies

To examine the therapeutic implications of our in vitro findings, we investigated the efficacy of combined SMAD1 inhibitor, DM, plus BTZ in a preclinical model of drug resistant MM. To this end, we established a mouse xenograft model with OPM2 vel/R in SCID mice and administered DM and BTZ alone or in combination. Notably, the combination of BTZ and DM suppressed tumor growth as compared with DM treatment alone, BTZ alone, or vehicle-alone (Fig. 6a). Importantly, the combination treatment significantly prolonged survival of mice without any untoward toxicity as indicated by body weight measurement (Fig. 6b and c). In addition, the level of p-SMAD1 in mouse tumors was reduced in the DM treated groups compared with control; as well as NF-κB1, TNFAIP8 and ID1 expression was reduced, and cleaved caspase 8 increased (Fig. 6d).

IHC analysis of tumor sections showed that treatment with BTZ plus DM resulted in reduced proliferation (Ki67 staining) and increased apoptosis (TUNEL analysis), compared to either BTZ or DM treatment alone (Fig. S6). These findings indicate that SMAD1 inhibition sensitizes MM cells to BTZ, promotes apoptosis, and reduces MM tumor growth in vivo.

## Discussion

In this study, we demonstrate that SMAD1 is an unfavorable prognostic biomarker for MM. To the best of our knowledge, this is the first study to demonstrate such a role of SMAD1 in MM. Our findings are consistent with studies on solid tumors in which upregulation of SMAD1 has been associated with tumor aggressiveness and poor outcomes [24]. Interestingly, high SMAD1 level could activate S1PR2 expression and induce apoptosis in diffuse large B-cell lymphoma [25]. This implies a context-or tumor-dependent expression and clinical significance for MM. Thus, targeting SMAD1 could be a potential therapeutic strategy for MM. Furthermore, SMAD1 may be a valuable biomarker for detecting the potential of drug resistance and relapse in MM patients.

SMAD1 is best characterized for activating ID1 [26, 27], which promotes proliferation and confers drug resistance in hepatocellular carcinoma [28], colon cancer [29], lung cancer [30], and acute myeloid leukemia [31]. However, whether SMAD1 exerts similar function in MM remains unclear. In our studies, we showed that SMAD1 regulates ID1 in MM. Furthermore, decreased expression of ID1 reduced MM cell proliferation and promoted apoptosis by activating p21 and p27. Of importance, we found that SMAD1 affects drug resistance and survival of MM cells through physical regulation NF-κB1 and increase TNFAIP8 expression. TNFAIP8 is known to counteract apoptosis by inhibiting caspase-8 activity, and by modulating other oncogenic targets such as growth factor receptors (EGFR and VEGFR), and cell cycle protein (Cyclin D1, phospho-Rb) [32, 33]. We previously demonstrated that TNFAIP8 overexpression was associated with MM cell drug resistance and proliferation [23]. This phenomenon has been described in lung cancer and cervical cancer [34, 35]. Several studies have reported that TNFAIP8 is induced by NF-κB1, inhibits cellular apoptosis, acts as an oncogene, and promotes cell growth/proliferation in human cancers [36, 37]. Here we show that SMAD1 could indirectly regulate TNFAIP8 through a crosstalk with NF-κB1.

In the analysis of public datasets, we found that SMAD1 expression in MM patient samples positively correlated with NF-κB1 expression. This phenomenon can be explained by our finding of SMAD1 binding sites in the proximal region of the NF-κB1 gene. The analysis employing the ENCODE data revealed 16 putative SMAD1 binding sites and significant enrichment of SMAD1 binding on the NF-κB1 promoter. In addition, we showed that, SMAD1 could affect expression level of NF-κB1 through IKKε and IκBα.

**Fig. 7:**
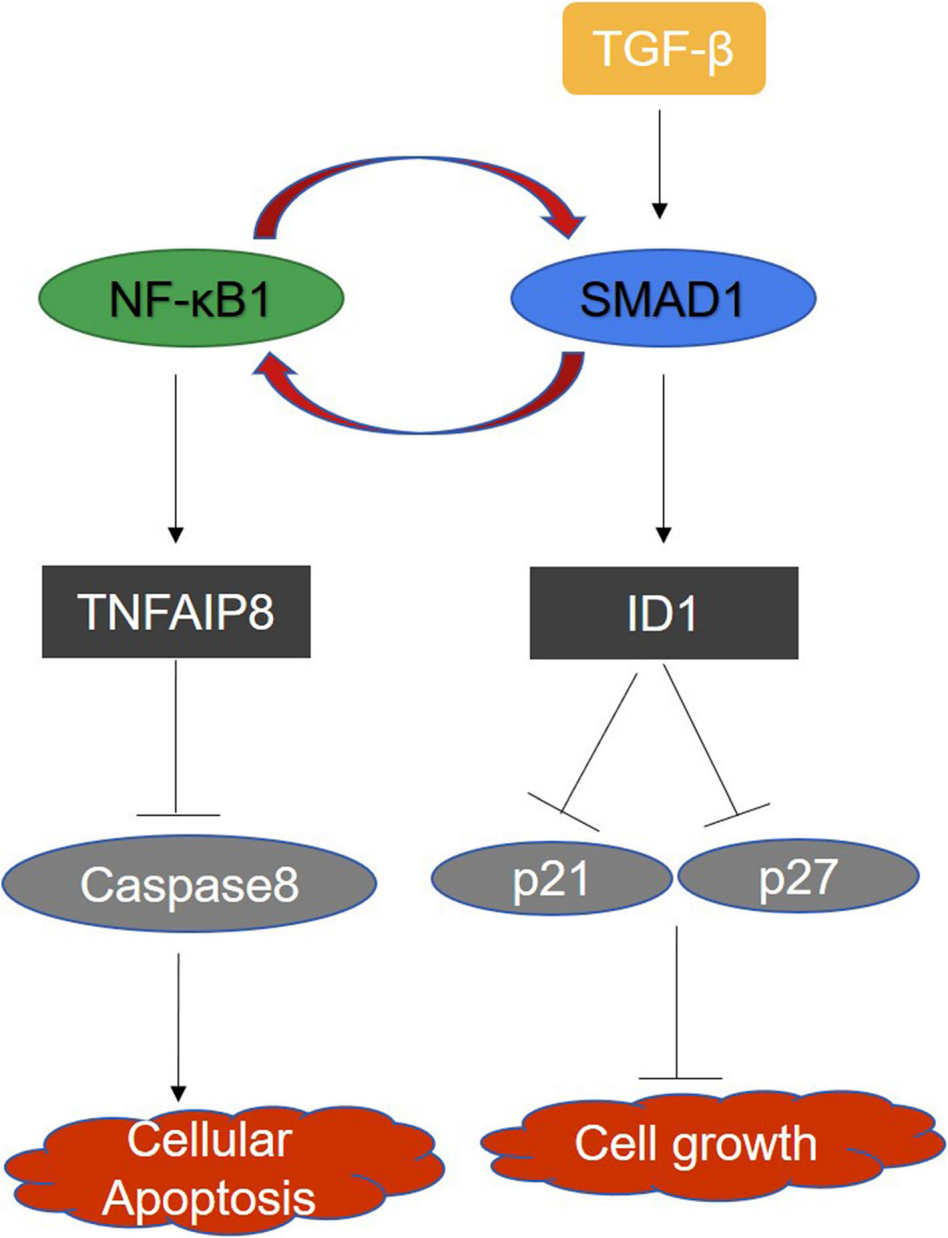
Proposed model for the effects of SMAD1 on apoptosis and cell growth in MM. SMAD1 inhibition affects growth through two axes: it blocks the cell cycle. Through ID1-p21/p27 and cell survival through NF-kB-TNFAIP8, the latter of which is critical in the context of drug resistant MM

The NF-κB transcription factor is known to affect the expression of more than 150 genes related to inflammation, cell proliferation, differentiation, and apoptosis [38]. Dysregulated NF-κB signaling has been observed in many hematological malignancies such as Hodgkin’s lymphoma, diffuse large B-cell lymphoma and MM [39–41]. However, several studies report that SMAD1 interacts with NF-κB1 in overexpression experiments in 293 T cells, and that SMAD1 bound selectively to endogenous NF-κB1 p50 protein in RAW 264.7 cells [42]. Consistent with these observation, it has been reported that the NF-κB1 p50 homodimer can regulate specific gene expression [43–45]. In our current study of MM, we found that SMAD1 expression is reduced after NF-κB1 knockdown, and vice versa, suggesting that SMAD1 may contribute to MM progression by activation of NF-κB1. In addition to activation of the ID1 pathway, a crosstalk between SMAD1 and NF-κB1 acting as a tumor inducer circuit, may provide a novel mechanism for SMAD1-mediated myelomagenesis (Fig. 7).

As drug-resistant MM cells are characterized by upregulation of both total and phosphorylated SMAD1, targeting either expression or phosphorylation of SMAD1 may represent a promising new approach to overcome MM drug-resistance. DM inhibits BMP signals by selectively inhibiting of BMP type I receptors ALK2, ALK3 and ALK6, resulting in suppression of SMAD1 phosphorylation. DM is known to induce cancer cell apoptosis [46]. In this study, we observed a clear dose-dependent cytotoxicity by DM in drug-resistant MM cells. Consistent with the cytotoxic effects in cell assays, phosphorylation of SMAD1 was also strongly inhibited by DM, suggesting inhibition of SMAD1 phosphorylation as a new modality to overcome drug-resistance in MM. Importantly, through in vitro and in vivo studies, we demonstrated that the combination of DM and BTZ significantly enhanced the inhibitory effect of these two drugs on MM cell viability, promoted MM cell apoptosis, and inhibited tumor growth. In contrast, no significant deleterious effects of DM treatment of DM plus BTZ combination treatment was observed on normal PBMCs from healthy donors, highlighting the specificity and safety of using such an approach to treat drug-resistant MM patients. This study provides proof-of-concept for the use of DM with BTZ in clinical practice.

## Conclusions

Our findings underscore the importance of SMAD1 as a determinant of drug resistance in MM cells through across talk with NF-κB1, suggesting a SMAD1-based targeting strategy to overcome MM drug resistance. Our results also indicate that DM is a potent anti-myeloma agent by inhibiting SMAD1 phosphorylation and may provide novel therapeutic strategies to treat MM.

## Supplementary Information

**Additional file 1: Supplementary materials and methods. Fig. S1**. Downregulated SMAD1 reduced migratory and invasive abilities of MM cells in vitro (A) Representative images of soft agar colony formation assays with the different cell models. Bar graphs indicate the colony count expressed in different microscopic fields. (B) Migration ability toward serum of 8226R5 and OPM2 vel/R cell lines were assessed using Transwell filter. Cells were pre-treated with DM 5uM for 24 h prior to the assay. (C) Migration ability toward serum of 8226R5 and OPM2 vel/R cell lines were assessed using Transwell filter. Cells were pre-treated with siSMAD1 50 nM and/or TGF-β 24 h Data were presented as mean ± s.d. from at least separate experiment. *: *p* < 0.05; **:*p* < 0.01; ***:p,0.001; **Fig. S2**. Correlation of SMAD1 expression with apoptosis activity (A) Correlation analysis of SMAD1 expression on the y axis and the Z-score enrichment across over 1000 cell lines from CCLE database. A Z-score was generated for each cell line in KEGG canonical pathway gene sets: apoptosis. Red circles indicate MM cell lines, and black circles indicate all CCLE cell lines except MM. A significant correlation between SMAD1 expression and the apoptosis activation was observed; *R* = − 0.1486, *p* < 0.0001.(B-F) Correlation between SMAD1 expression and apoptosis activation was also observed in other hematological malignancies. **Fig. S3**. SMAD1 inhibition induces apoptosis in MM cells. (A) OPM2 vel/R, MM1.R cell lines were treated with 10uM of either DM or DMSO control for 24 h, treated with 10 nM BTZ or vehicle for 48 h, and then subjected to annexin-V/propidium iodide analysis by flow cytometry to determine percentage of apoptotic cells(left). (B) OPM2 vel/R, MM1.R cells lines were transfected with 30 nM of either siSMAD1 or siRNA control for 24 h, treated with 10 nM BTZ or vehicle for 48 h, and subjected to annexin-V/propidium iodide analysis to determine percentage of apoptotic cells. **Fig. S4.** Combination of drugs (Dox, Dex, Len, DM) synergistically induces cytotoxic effects on primary MM patients’ sample. (A) Primary mononuclear cells derived from 5 MM patients were treated with indicated concentration of Dox, Dex, Len and DM for 48 h, and then cell viabiility was evaluated by MTT assays. (B) CD138 + cells derived from 2 MM patients were treated with indicated concentration of Dex, Dox, Len and DM for 48 h, and then cell viabiility was evaluated by MTT assays. (C) PBMCs derived from three healthy donors were treated with indicated concentrations of DM alone or in the presence of 10 nM BTZ for 48 h and cytotoxicity was assessed by MTT. Results are presented as mean ± s.d. from at least separate experiment. *: *p* < 0.05; **:*p* < 0.01; ***:p,0.001; NS: not significant. **Fig. S5** (A)SMAD1 binding sties and histone modification around the NF-κB1 promoter identified by Chip-seq data from ENCODE. (B) Genome-wide identification of transcription binding sites of SMAD1 in the NF-κB1 promoter region based on UCSC data. (C) 16 putative sites were predicted with these setting (80%) in sequence named hg38_genscan_chr4.1488. **Fig. S6**. Representative microscopic images of tumor sections from four treated groups analyzed for histology (H&E), proliferation (Ki-67) or apoptosis (TUNEL). **STable 1**. The top 5 KEGG pathway enriched by GSEA in APEX trial dataset by comparing the SMAD1-high and SMAD1-low expression samples. **Fig. S7 (A)** In parallel with cell cycle assay, western blot was performed to measure the efficiency of siRNA in MM cell lines. (B) In parallel with immunofluorescence, Protein lysate was subjected to western blot with indicated antibodies.

## Data Availability

Available upon request.

## Abbreviations

MM: Multiple myeloma
SMAD1: Small Body Size (SMA) and Mothers Against Decapentaplegic family 1
DM: Dorsomorphin
TNFAIP8: TNFα-Induced protein 8
BTZ: Bortezomib
DEX: Dexamethasone
p-SMAD1: SMAD1 phosphorylation
BMPs: Bone morphogenetic proteins
GEO: Gene Expression Omnibus
ND: Normal donor
PFS: Progression-free survival
OS: Overall survival
ROC: Receiver operating characteristic
AUC: Area under the curve
GSEA: Gene set enrichment analysis
CCLE: Cancer Cell Line Encyclopedia
Len: Lenalidomide
Dox: Doxorubicin
ENCODE: The Encyclopedia of DNA Elements
ChiP: Chromatin immunoprecipitation

## References

[1] Sonneveld P, Broijl A (2016). Treatment of relapsed and refractory multiple myeloma. Haematologica.

[2] Kirshner J, Thulien KJ, Martin LD, Debes Marun C, Reiman T, Belch AR (2008). A unique three-dimensional model for evaluating the impact of therapy on multiple myeloma. Blood.

[3] Chaudhury A, Howe PH (2009). The tale of transforming growth factor-beta (TGFbeta) signaling: a soigne enigma. IUBMB Life.

[4] Liu X, Yue J, Frey RS, Zhu Q, Mulder KM (1998). Transforming growth factor beta signaling through Smad1 in human breast cancer cells. Cancer Res.

[5] Olsen OE, Hella H, Elsaadi S, Jacobi C, Martinez-Hackert E, Holien T. Activins as dual specificity TGF-beta family molecules: SMAD-activation via Activin- and BMP-type 1 receptors. Biomolecules. 2020;10(4) e-pub ahead of print 2020/04/03. 10.3390/biom10040519. 10.3390/biom10040519 PMC7225989 32235336

[6] Nickel J, Mueller TD. Specification of BMP signaling. Cells. 2019;8(12) e-pub ahead of print 2019/12/11. 10.3390/cells8121579. 10.3390/cells8121579 PMC6953019 31817503

[7] Chandrasinghe P, Cereser B, Moorghen M, Al Bakir I, Tabassum N, Hart A (2018). Role of SMAD proteins in colitis-associated cancer: from known to the unknown. Oncogene.

[8] Nokin MJ, Bellier J, Durieux F, Peulen O, Rademaker G, Gabriel M (2019). Methylglyoxal, a glycolysis metabolite, triggers metastasis through MEK/ERK/SMAD1 pathway activation in breast cancer. Breast Cancer Res.

[9] Eikesdal HP, Becker LM, Teng Y, Kizu A, Carstens JL, Kanasaki K (2018). BMP7 signaling in TGFBR2-deficient stromal cells provokes epithelial carcinogenesis. Mol Cancer Res.

[10] Jung JW, Yoon SM, Kim S, Jeon YH, Yoon BH, Yang SG (2016). Bone morphogenetic protein-9 is a potent growth inhibitor of hepatocellular carcinoma and reduces the liver cancer stem cells population. Oncotarget.

[11] Liu X, Guo H, Wei Y, Cai C, Zhang B, Li J (2017). TGF-beta induces growth suppression in multiple myeloma MM.1S cells via E2F1. Oncol Lett.

[12] Wen J, Tao W, Kuiatse I, Lin P, Feng Y, Jones RJ (2015). Dynamic balance of multiple myeloma clonogenic side population cell percentages controlled by environmental conditions. Int J Cancer.

[13] Wang D, Hao C, Zhang L, Zhang J, Liu S, Li Y (2020). Exosomal miR-125a-5p derived from silica-exposed macrophages induces fibroblast transdifferentiation. Ecotoxicol Environ Saf.

[14] Jin DH, Kim Y, Lee BB, Han J, Kim HK, Shim YM (2017). Metformin induces cell cycle arrest at the G1 phase through E2F8 suppression in lung cancer cells. Oncotarget.

[15] Reeves ME, Firek M, Chen ST, Amaar YG (2014). Evidence that RASSF1C stimulation of lung cancer cell proliferation depends on IGFBP-5 and PIWIL1 expression levels. PLoS One.

[16] Buzzeo R, Enkemann S, Nimmanapalli R, Alsina M, Lichtenheld MG, Dalton WS (2005). Characterization of a R115777-resistant human multiple myeloma cell line with cross-resistance to PS-341. Clin Cancer Res.

[17] Yang Y, Chen Y, Saha MN, Chen J, Evans K, Qiu L (2015). Targeting phospho-MARCKS overcomes drug-resistance and induces antitumor activity in preclinical models of multiple myeloma. Leukemia.

[18] Saha MN, Chen Y, Chen MH, Chen G, Chang H (2014). Small molecule MIRA-1 induces in vitro and in vivo anti-myeloma activity and synergizes with current anti-myeloma agents. Br J Cancer.

[19] Li F, Yao FS, Zhu XJ, Gu WY, Wang XH, Chen B (2019). A randomized phase II, open-label and multicenter study of combination regimens of bortezomib at two doses by subcutaneous injection for newly diagnosed multiple myeloma patients. J Cancer Res Clin Oncol.

[20] Singh SK, Singh S, Gadomski S, Sun L, Pfannenstein A, Magidson V (2018). Id1 ablation protects hematopoietic stem cells from stress-induced exhaustion and aging. Cell Stem Cell.

[21] Beyreis M, Gaisberger M, Jakab M, Neureiter D, Helm K, Ritter M, et al. The cancer stem cell inhibitor napabucasin (BBI608) shows general cytotoxicity in biliary tract cancer cells and reduces cancer stem cell characteristics. Cancers (Basel). 2019;11(3) e-pub ahead of print 2019/03/01. 10.3390/cancers11030276. 10.3390/cancers11030276 PMC6468451 30813586

[22] Niture S, Ramalinga M, Kedir H, Patacsil D, Niture SS, Li J (2018). TNFAIP8 promotes prostate cancer cell survival by inducing autophagy. Oncotarget.

[23] Rastgoo N, Wu J, Liu M, Pourabdollah M, Atenafu EG, Reece D, et al. Targeting CD47/TNFAIP8 by miR-155 overcomes drug resistance and inhibits tumor growth through induction of phagocytosis and apoptosis in multiple myeloma. Haematologica. 2019. e-pub ahead of print 2019/11/30. 10.3324/haematol.2019.227579. 10.3324/haematol.2019.227579 PMC7716364 33256380

[24] Ruan X, Zuo Q, Jia H, Chau J, Lin J, Ao J (2015). P53 deficiency-induced Smad1 upregulation suppresses tumorigenesis and causes chemoresistance in colorectal cancers. J Mol Cell Biol.

[25] Stelling A, Hashwah H, Bertram K, Manz MG, Tzankov A, Muller A (2018). The tumor suppressive TGF-beta/SMAD1/S1PR2 signaling axis is recurrently inactivated in diffuse large B-cell lymphoma. Blood.

[26] Chen T, Heller E, Beronja S, Oshimori N, Stokes N, Fuchs E (2012). An RNA interference screen uncovers a new molecule in stem cell self-renewal and long-term regeneration. Nature.

[27] Yu H, Yue X, Zhao Y, Li X, Wu L, Zhang C (2014). LIF negatively regulates tumour-suppressor p53 through Stat3/ID1/MDM2 in colorectal cancers. Nat Commun.

[28] Niu LL, Cheng CL, Li MY, Yang SL, Hu BG, Chong CCN (2018). ID1-induced p16/IL6 axis activation contributes to the resistant of hepatocellular carcinoma cells to sorafenib. Cell Death Dis.

[29] Przybyla T, Sakowicz-Burkiewicz M, Maciejewska I, Bielarczyk H, Pawelczyk T (2017). Suppression of ID1 expression in colon cancer cells increases sensitivity to 5-fluorouracil. Acta Biochim Pol.

[30] Castanon E, Bosch-Barrera J, Lopez I, Collado V, Moreno M, Lopez-Picazo JM (2013). Id1 and Id3 co-expression correlates with clinical outcome in stage III-N2 non-small cell lung cancer patients treated with definitive chemoradiotherapy. J Transl Med.

[31] Battula VL, Le PM, Sun JC, Nguyen K, Yuan B, Zhou X, et al. AML-induced osteogenic differentiation in mesenchymal stromal cells supports leukemia growth. JCI Insight. 2017;2(13) e-pub ahead of print 2017/07/07. 10.1172/jci.insight.90036. 10.1172/jci.insight.90036 PMC5499365 28679949

[32] Padmavathi G, Banik K, Monisha J, Bordoloi D, Shabnam B, Arfuso F (2018). Novel tumor necrosis factor-alpha induced protein eight (TNFAIP8/TIPE) family: Functions and downstream targets involved in cancer progression. Cancer Lett.

[33] Niture S, Dong X, Arthur E, Chimeh U, Niture SS, Zheng W, et al. Oncogenic role of tumor necrosis factor alpha-induced protein 8 (TNFAIP8). Cells. 2018;8(1) e-pub ahead of print 2018/12/28. 10.3390/cells8010009. 10.3390/cells8010009 PMC6356598 30586922

[34] Xing Y, Liu Y, Liu T, Meng Q, Lu H, Liu W (2018). TNFAIP8 promotes the proliferation and cisplatin chemoresistance of non-small cell lung cancer through MDM2/p53 pathway. Cell Commun Signal.

[35] Wu S, Li W, Wu Z, Cheng T, Wang P, Li N (2019). TNFAIP8 promotes cisplatin resistance in cervical carcinoma cells by inhibiting cellular apoptosis. Oncol Lett.

[36] Afrasiabi A, Parnell GP, Fewings N, Schibeci SD, Basuki MA, Chandramohan R (2019). Evidence from genome wide association studies implicates reduced control of Epstein-Barr virus infection in multiple sclerosis susceptibility. Genome Med.

[37] Li Y, Jing C, Chen Y, Wang J, Zhou M, Liu X (2015). Expression of tumor necrosis factor alpha-induced protein 8 is upregulated in human gastric cancer and regulates cell proliferation, invasion and migration. Mol Med Rep.

[38] Sabir JSM, El Omri A, Shaik NA, Banaganapalli B, Al-Shaeri MA, Alkenani NA (2019). Identification of key regulatory genes connected to NF-kappaB family of proteins in visceral adipose tissues using gene expression and weighted protein interaction network. PLoS One.

[39] Cai Q, Tu M, Xu-Monette ZY, Sun R, Manyam GC, Xu X (2017). NF-kappaB p50 activation associated with immune dysregulation confers poorer survival for diffuse large B-cell lymphoma patients with wild-type p53. Mod Pathol.

[40] Sud A, Cooke R, Swerdlow AJ, Houlston RS (2015). Genome-wide homozygosity signature and risk of Hodgkin lymphoma. Sci Rep.

[41] Allegra A, Speciale A, Molonia MS, Guglielmo L, Musolino C, Ferlazzo G (2018). Curcumin ameliorates the in vitro efficacy of carfilzomib in human multiple myeloma U266 cells targeting p53 and NF-kappaB pathways. Toxicol In Vitro.

[42] Kwon SJ, Lee GT, Lee JH, Iwakura Y, Kim WJ, Kim IY (2014). Mechanism of pro-tumorigenic effect of BMP-6: neovascularization involving tumor-associated macrophages and IL-1a. Prostate.

[43] Smith EL, Somma D, Kerrigan D, McIntyre Z, Cole JJ, Liang KL (2019). The regulation of sequence specific NF-kappaB DNA binding and transcription by IKKbeta phosphorylation of NF-kappaB p50 at serine 80. Nucleic Acids Res.

[44] Markopoulos GS, Roupakia E, Tokamani M, Alabasi G, Sandaltzopoulos R, Marcu KB, et al. Roles of NF-kappaB signaling in the regulation of miRNAs impacting on inflammation in cancer. Biomedicines. 2018;6(2) e-pub ahead of print 2018/03/31. 10.3390/biomedicines6020040. 10.3390/biomedicines6020040 PMC6027290 29601548

[45] Kumar D, Lee B, Puan KJ, Lee W, Luis BS, Yusof N (2019). Resistin expression in human monocytes is controlled by two linked promoter SNPs mediating NFKB p50/p50 binding and C-methylation. Sci Rep.

[46] Aikawa A, Kozako T, Uchida Y, Yoshimitsu M, Ishitsuka K, Ohsugi T, et al. Cell death induced by dorsomorphin in adult T-cell leukemia/lymphoma is AMPK-independent. FEBS J. 2020. e-pub ahead of print 2020/02/07. 10.1111/febs.15239. 10.1111/febs.15239 32027454

