# Supplementary material for: SMAD1 as a biomarker and potential therapeutic target in drug-resistant multiple myeloma
Source: Biomark Res. 2021 Jun 16;9:48. doi: 10.1186/s40364-021-00296-7 (PMC8207655; doi:10.1186/s40364-021-00296-7)
Supplement: Supplementary file 1 — Additional file 1: Supplementary materials and methods. Fig. S1. Downregulated SMAD1 reduced migratory and invasive abilities of MM cells in vitro (A) Representative images of soft agar colony formation assays with the different cell models. Bar graphs indicate the colony count expressed in different microscopic fields. (B) Migration ability toward serum of 8226R5 and OPM2 vel/R cell lines were assessed using Transwell filter. Cells were pre-treated with DM 5uM for 24 h prior to the assay. (C) Migration ability toward serum of 8226R5 and OPM2 vel/R cell lines were assessed using Transwell filter. Cells were pre-treated with siSMAD1 50 nM and/or TGF-β 24 h Data were presented as mean ± s.d. from at least separate experiment. *: p < 0.05; **:p < 0.01; ***:p,0.001; Fig. S2. Correlation of SMAD1 expression with apoptosis activity (A) Correlation analysis of SMAD1 expression on the y axis and the Z-score enrichment across over 1000 cell lines from CCLE database. A Z-score was generated for each cell line in KEGG canonical pathway gene sets: apoptosis. Red circles indicate MM cell lines, and black circles indicate all CCLE cell lines except MM. A significant correlation between SMAD1 expression and the apoptosis activation was observed; R = − 0.1486, p < 0.0001.(B-F) Correlation between SMAD1 expression and apoptosis activation was also observed in other hematological malignancies. Fig. S3. SMAD1 inhibition induces apoptosis in MM cells. (A) OPM2 vel/R, MM1.R cell lines were treated with 10uM of either DM or DMSO control for 24 h, treated with 10 nM BTZ or vehicle for 48 h, and then subjected to annexin-V/propidium iodide analysis by flow cytometry to determine percentage of apoptotic cells(left). (B) OPM2 vel/R, MM1.R cells lines were transfected with 30 nM of either siSMAD1 or siRNA control for 24 h, treated with 10 nM BTZ or vehicle for 48 h, and subjected to annexin-V/propidium iodide analysis to determine percentage of apoptotic cells. Fig. S4. Combination of drugs ( [file 40364_2021_296_MOESM1_ESM.docx]

**Supplementary materials and methods**

**Drugs and reagents:**

Bortezomib, Selleck Chemicals, Houston TX 77230 USA;

Lenalidomide (CC-5013), Selleck Chemicals, Houston TX 77230 USA;

Dexamethasone Sodium Phosphate, Selleck Chemicals, Houston TX 77230 USA;

Doxorubicin (Adriamycin), Selleck Chemicals, Houston TX 77230 USA;

Dorsomorphin, Sigma, 086m4754v, Houston TX 77230 USA;

TGF-β1, Peptotech, Cat No 100-21-100UG, USA

All chemical agents were dissolved in dimethyl sulfoxide except special indication and further diluted in RPMI1640 medium.

**Antibodies for Western blotting:**

Anti-SMAD1, Cell Signaling Technology, Inc. Danvers, MA;

Anti-pSMAD1, Cell Signaling Technology, 9516T, Inc. Danvers, MA;

Anti-NFKB1, Cell Signaling Technology, Inc. Danvers, MA;

Anti-IKBα, Cell Signaling Technology, Danvers, MA;

Anti-IKKε, Cell Signaling Technology, Danvers, MA;

Anti-ID1, Proteintech, Inc. Danvers, MA;

Anti- Caspase-3, Cell Signaling Technology, Inc. Danvers, MA, USA;

Anti-Cleaved Caspase-3, Cell Signaling Technology, Inc. Danvers, MA, USA;

Anti-Cleaved Caspase-8(Asp384), Cell Signaling Technology, Inc. Danvers, MA, USA;

Anti-TNFAIP8, Elabscience, Inc. Danvers, MA, USA;

Anti-CDK6, SAB Signalway Antibody, Dallas, Texas USA;

Anti-bcl2, Santa Cruz Biotechnology, Dallas, Texas USA;

Anti-P21, Cell Signaling Technology, Inc. Danvers, MA;

Anti-P27, SAB Signalway Antibody, Inc. Danvers, MA;

Anti-GAPDH (Glyceraldehyde-3-phosphate dehydrogenase), Cell Signaling Technology, Inc. Danvers, MA

**Primers used in this study**

NK-κB1 promoter primers

Forward: 5’-TTTTCTGCCTCCACCTCTGT-3’

Reverse:5’-AGGAAGCCAAGGAGGCTAAA-3’

**Gene silencing and siRNA transfection**

MM cell lines were transiently transfected with either siSMAD1, siNFKB1, siID1 or a scrambled siRNA (Santa cruz) using liposome 3000 transfection reagent (Invitrogen, Catalog #L3000008) according to the manufacturer’s instructions. Functional experiments were performed following transfection. The transfected cells were examined for qPCR or cell viability using the 3-(4,5-dimethylthiozol-2-yl)-2,5-diphenyltetrazolium bromide (MTT; Biobasic) assay 48 h after transfection.

**Cell viability and apoptosis assays**

Cell viability was assessed by MTT colorimetric assay. MM cell lines were seeded in 96-well plates (Sarstedt, Inc.) in 100 μL complete medium at a density of 2.0 × 104 cells per well. MM cells were incubated at 37℃ and 5% CO2 with various concentrations of indicated drugs for 48 h. After the incubation, 10 µl of MTT (0.5 mg/mL) was added, and the cells were further incubated for an additional 4 h. Finally, precipitated dye was solubilized using acidified isopropanol and the absorbance of the wells was read through a microplate reader. Each experiment was performed in triplicate and the mean value and SD were calculated. To examine apoptotic cell death, MM cells were treated with various concentrations of BTZ in combination with siRNA or DM for 24 h followed by annexin V-FITC/PI staining for apoptosis analysis in a FACS Calibur flow cytometer (Becton Dickinson). Captured events were analyzed using CellQuest software. The extent of apoptosis was quantified as percentage of annexin-V positive cells, and the extent of drug-specific apoptosis was assessed by the formula: percentage of annexin-V positive cells = (test - control) × 100/ (100 - control).

**Protein extraction and western blotting**

Cells were lysed in cold cell lysis buffer (50 mmol/L Tris-HCl, pH 7.4, 150 mmol/L NaCl, 1% NP-40, 0.5% sodium deoxycholate, and 0.1% SDS) supplemented with protease inhibitor cocktail or directly in 6×SDS sample buffer (250 mM Tris-HCl pH 6.8, 30% (v/v) glycerol, 10% (w/v) SDS, 0.5 M DTT, and 0.012% (w/v) bromophenol blue). Equal amounts of proteins were resolved by 8-15% SDS-PAGE and transferred onto PVDF membranes. Membranes were blocked by incubation in 5% nonfat dry milk in TBST (0.05% Tween-20 in PBS) and probed with primary antibodies mentioned above. Blots were then developed by SuperSignal West Pico Chemiluminescent Substrate (Thermo Scientific). To evaluate protein levels in tumor tissue, whole protein extracts were prepared from 4 representative tumors of each group using T-PER lysis solution (Pierce). Briefly, 50 mg of tissue samples were resuspended in 400 μL of lysis buffer supplemented with protease inhibitors. Samples were centrifuged at 15,000*g* for 15 minutes, and 50 µg of each supernatant was then analyzed by SDS-PAGE for protein level analysis.

**Soft-agar colony formation, and migration assays**

For soft-agar assays, 500cells were suspended in RPMI1640 containing 1% agar and DM 1uM in each well. After growing at 37℃ for 21 days, colonies were visualized and counted in three different microscopic fields. Migration was performed using 24-well Transwell plate in the presence of 10% FBS. Fibronectin (Sigma-Aldrich) was added to the lower chamber (Greiner bio-one) in 24-well plates and the plates were maintained in a cell incubator at 37℃ for 30 mins. and cells that migrated to the lower chambers were labeled with crystal violet and counted in three different microscopic fields.

**Chromatin immunoprecipitation (Chip)**

For ChIP Analysis, a total of 5 million treated cells were treated in culture with formaldehyde to a ﬁnal concentration of 1% for 10 minutes at 37°C. Cells were harvested by centrifugation, washed with 2 x 10 mL PBS, and chromatin was immunoprecipitated as described in manufacture manual. Acetylated histone H3 antibody (Cell Signaling Technology) or targets antibody (Cell Signaling Technology) was used. After precipitation, 2 ng DNA was used for each real-time PCR. Quantitation was performed using SYBR Green on an ABI7500 TaqMan machine (40 cycles) using self-designed primers that had been tested for lack of primer-dimer artifacts and for single-species ampliﬁcation. Values for immunoprecipitations were normalized to input DNA values, giving data in arbitrary units.

**Confocal laser scanning microscopy**
RPMI8226-R5 cells and OPM2 vel/R were allowed to attach to a glass slide coated with 10 µg/mL fibronectin (Sigma) for 1 hour in 37℃. Cells were subsequently fixated using 4% formaldehyde in PBS for 15 minutes at room temperature. After fixation, slides were blocked with 10% FBS in cell culture medium and subsequently incubated overnight at 4°C with SMAD1 antibody (sc-7965, Santa Cruz) and NF-κB1 antibody (sc-166588, Santa Cruz). Slides were subsequently washed three times with PBS and stained with goat-anti-mouse secondary antibody for 1 hour. After three PBS washes, slides were stained with DAPI (#4083, cell signaling) for 5 minutes and mounted with mounting medium (Vector Laboratories Inc). Images were acquired using a confocal laser scanning microscope (Leica SP8). Sequential scanning of different channels was performed at a resolution of 512 x 512 pixels.

The system was equipped with a HC PLAPO CS2 40×1.1. WATER objective. NF-κB1 and SMAD1 were excited with a Diode 488-nm laser and a Diode 594-nm laser respectively. Brightness were optimized and applied to the entire image.

**Myeloma xenograft mouse model**

Immunodeficient (SCID) mice (male, 6–8 weeks old; OCI) were housed in the animal care facility, under a 12/12 hours light/dark cycle at 22°C, and they received a standard diet and acidified water ad libitum. Using a protocol approved by the animal testing ethical committee of the UHN, mice were inoculated subcutaneously at their lower dorsum with 1×10^7^ 8226-R5 cells in matrigel basement membrane matrix (Becton Dickinson). When tumors were palpable (approximately 21 days after injection), mice were randomly assigned into 4 groups (n=4 in each), receiving intraperitoneal injection twice a week with 0.5 mg/kg BTZ alone or combined with DM 10mg/kg, DM alone or an equal volume of vehicle in 3 days interval for 15 days. The shortest and longest diameters of the tumor were measured with external calipers every 3 days, and tumor volume (in mm3) was calculated using the following standard formula: V = 0.5a x b2, where “a” and “b” are the long and short diameter of the tumor. Survival was evaluated from the first day of tumor injection until death. In accordance with institutional guidelines, mice were sacrificed when their tumors reached 1.5 cm in diameter or in the event of paralysis or major compromise in their quality of life, to prevent unexpected suffering. Immunohistochemical studies were performed on formalin-fixed Tumor samples using H & E, Ki-67 and TUNEL (terminal deoxynucleotidyl transferase mediated dUTP nick end labeling) staining.

**Statistical analysis**

Categorical variables such as SMAD1 status, gender and vital status will be summarized with counts and percentages. The Continuous variable age at diagnosis and follow-up will be summarized with medians and ranges. Overall Survival (OS) and Progression Free Survival (PFS) rates were calculated using the Kaplan-Meier product-limit method. Log-rank test was used as a univariate analysis to compare levels of patient characteristics and other potential predictive factors. All P-values were 2-sided and for the statistical analyses and P < 0.05 will be considered to indicate a significantly different result. Statistical analyses will be performed using SAS Version 9.4 (2002-2012 SAS Institute Inc., Cary, NC, USA).

All quantified data represent a mean of triplicate experiments **±** SEM which analyzed with GraphPad Prism 5 (GraphPad Software, La Jolla, CA) and comparisons between different groups were assessed by the Student’s ***t*** test and one-way analysis of variance. The correlation between SMAD1 and NF-κB1 expression was determined using Pearson’s coefficient test. Survival curves were estimated using Kaplan–Meier method and log-rank test was used to compute differences between the curves. Differences were considered significant at values of P**<**0.05.


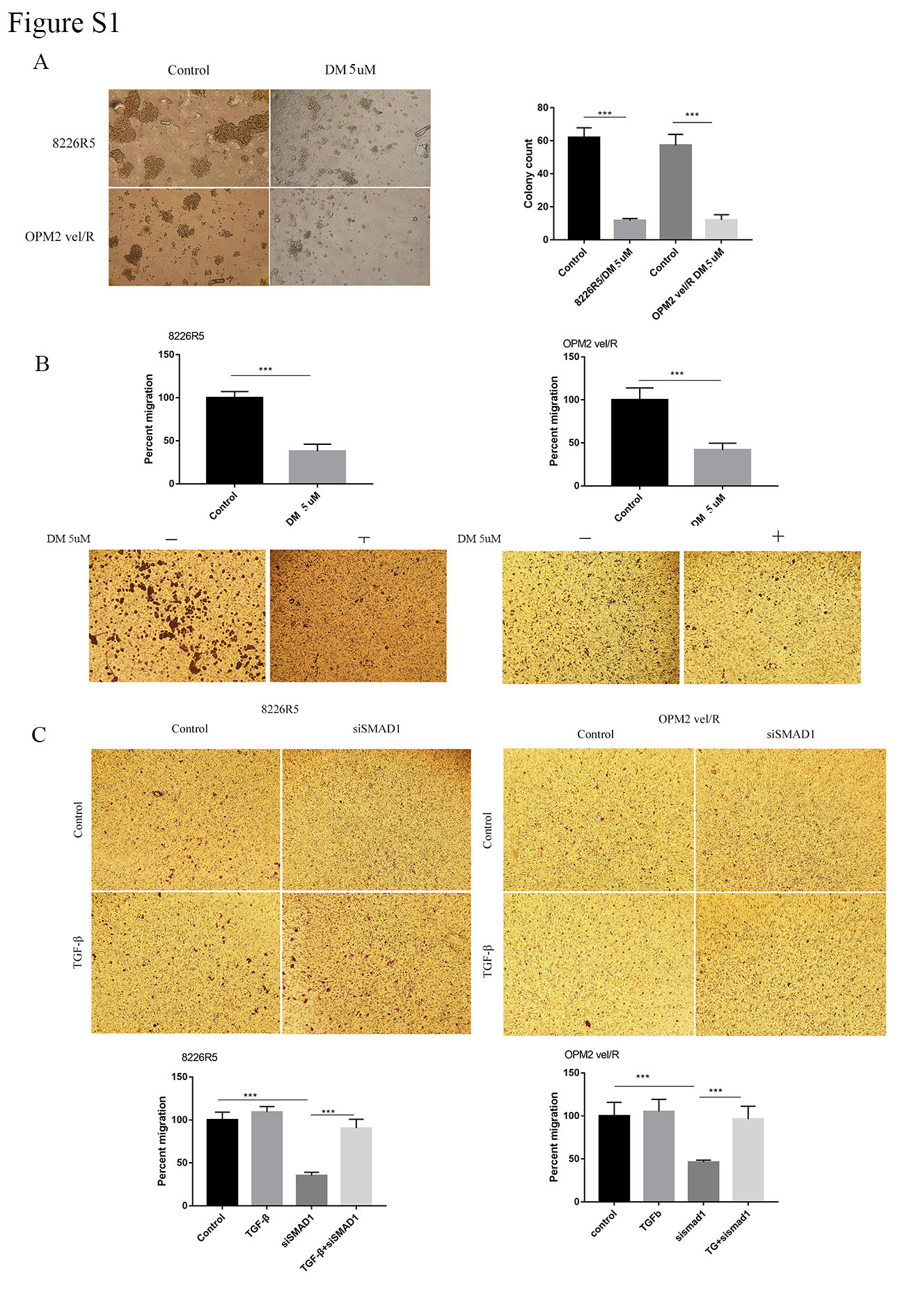


**Fig S1. Downregulated SMAD1 reduced migratory and invasive abilities of MM cells in vitro** (A) Representative images of soft agar colony formation assays with the different cell models. Bar graphs indicate the colony count expressed in different microscopic fields. (B) Migration ability toward serum of 8226R5 and OPM2 vel/R cell lines were assessed using Transwell filter. Cells were pre-treated with DM 5uM for 24h prior to the assay. (C) Migration ability toward serum of 8226R5 and OPM2 vel/R cell lines were assessed using Transwell filter. Cells were pre-treated with siSMAD1 50nM and/or TGF-β 24h Data were presented as mean ± s.d. from at least separate experiment. *: p<0.05; **:p<0.01; ***:p,0.001;


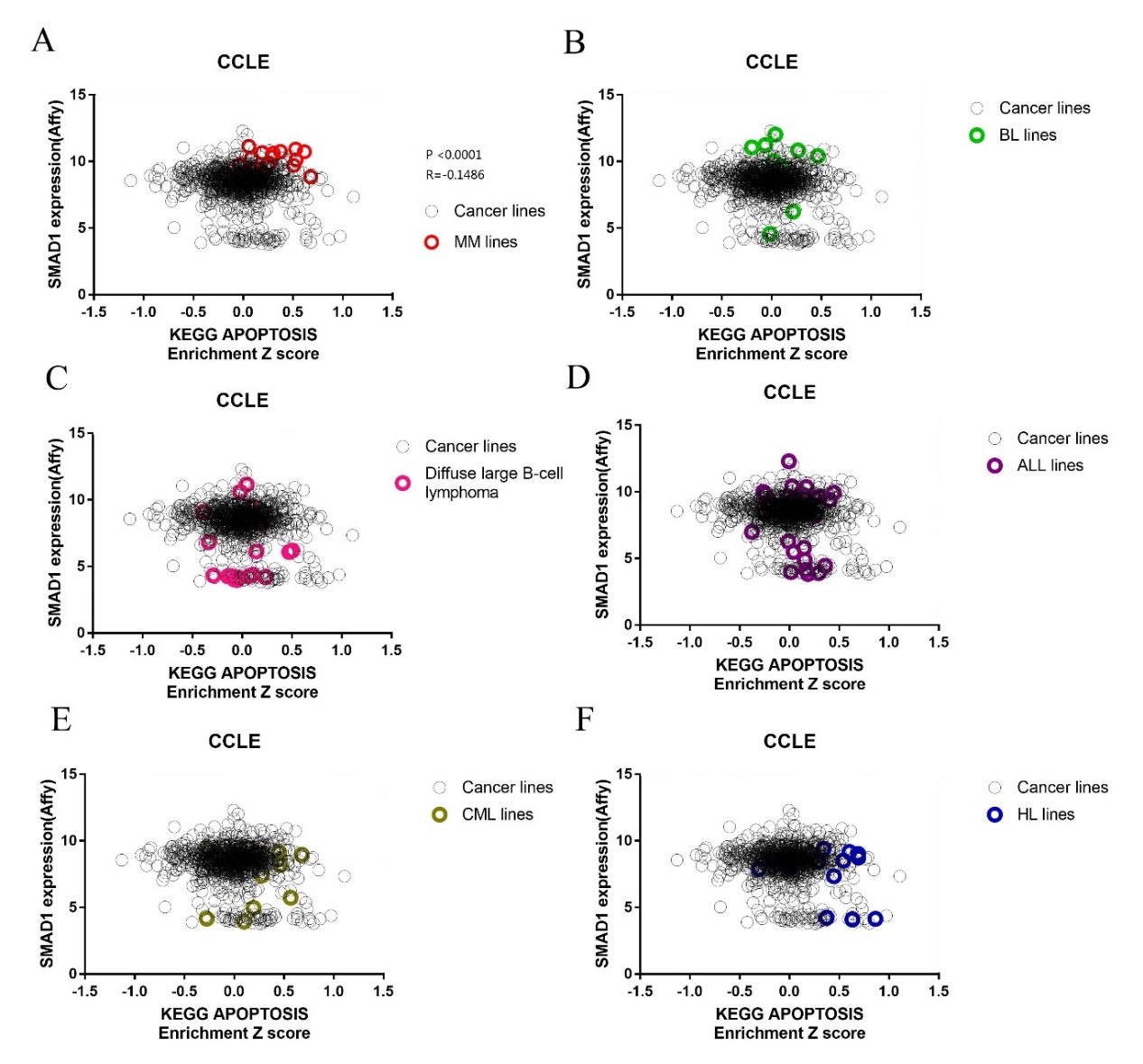


**Fig S2. Correlation of SMAD1 expression with apoptosis activity** (A) Correlation analysis of SMAD1 expression on the y axis and the Z-score enrichment across over 1000 cell lines from CCLE database. A Z-score was generated for each cell line in KEGG canonical pathway gene sets: apoptosis. Red circles indicate MM cell lines, and black circles indicate all CCLE cell lines except MM. A significant correlation between SMAD1 expression and the apoptosis activation was observed; R= -0.1486, p<0.0001.(B-F) Correlation between SMAD1 expression and apoptosis activation was also observed in other hematological malignancies.


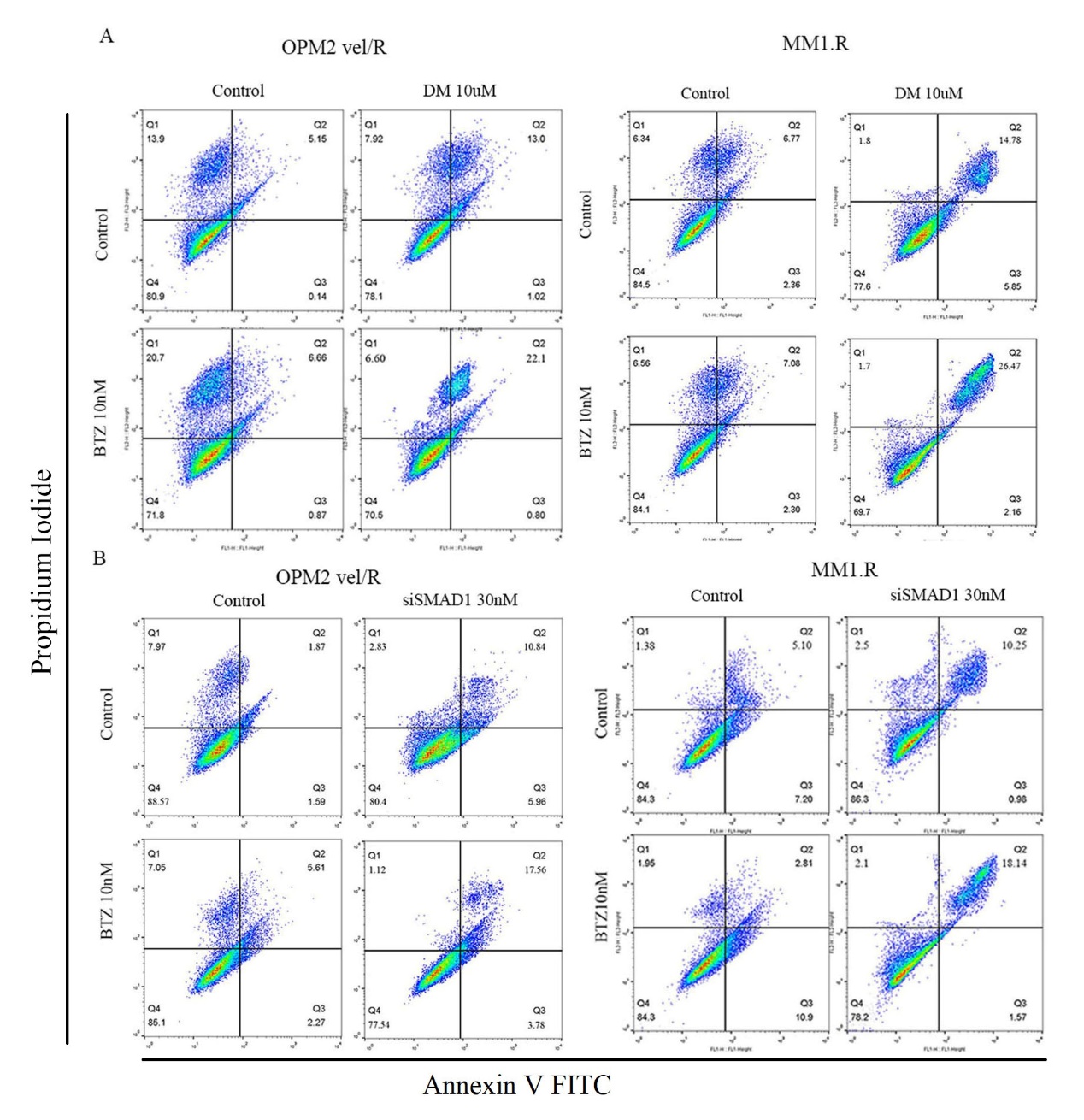


**Fig S3. SMAD1 inhibition induces apoptosis in MM cells.**

(A) OPM2 vel/R, MM1.R cell lines were treated with 10uM of either DM or DMSO control for 24h, treated with 10nM BTZ or vehicle for 48h, and then subjected to annexin-V/propidium iodide analysis by flow cytometry to determine percentage of apoptotic cells(left). (B) OPM2 vel/R, MM1.R cells lines were transfected with 30nM of either siSMAD1 or siRNA control for 24h, treated with 10nM BTZ or vehicle for 48h, and subjected to annexin-V/propidium iodide analysis to determine percentage of apoptotic cells.


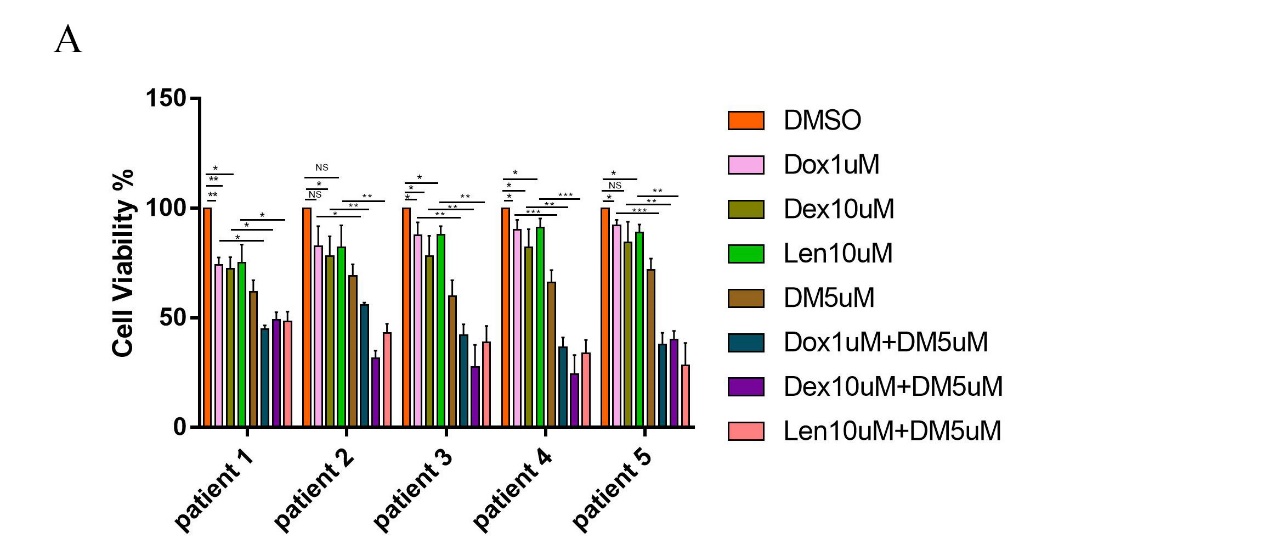


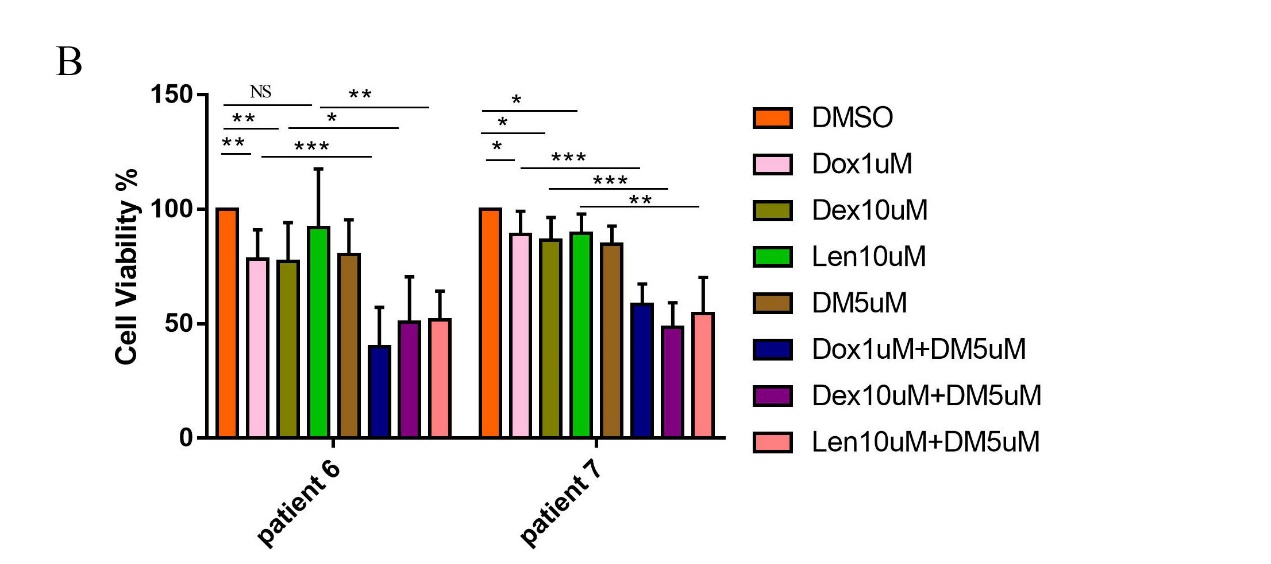


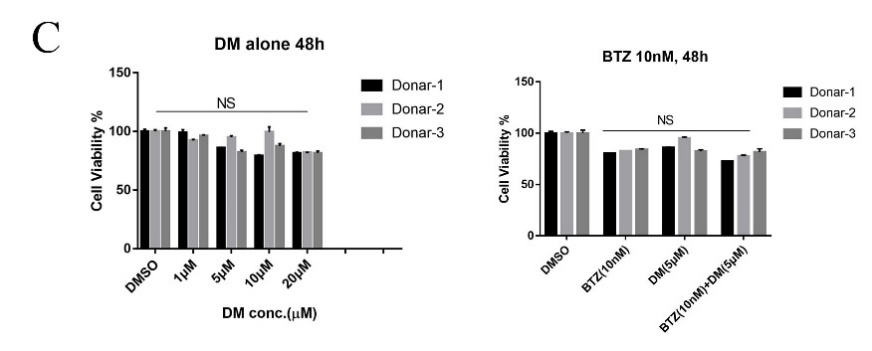


**Fig S4.** Combination of drugs (Dox, Dex, Len, DM) synergistically induces cytotoxic effects on primary MM patients’ sample. (A) Primary mononuclear cells derived from 5 MM patients were treated with indicated concentration of Dox, Dex, Len and DM for 48h, and then cell viabiility was evaluated by MTT assays. (B) CD138 + cells derived from 2 MM patients were treated with indicated concentration of Dex, Dox, Len and DM for 48h, and then cell viabiility was evaluated by MTT assays. (C) PBMCs derived from three healthy donors were treated with indicated concentrations of DM alone or in the presence of 10nM BTZ for 48h and cytotoxicity was assessed by MTT. Results are presented as mean ± s.d. from at least separate experiment. *: p<0.05; **:p<0.01; ***:p,0.001; NS: not significant.


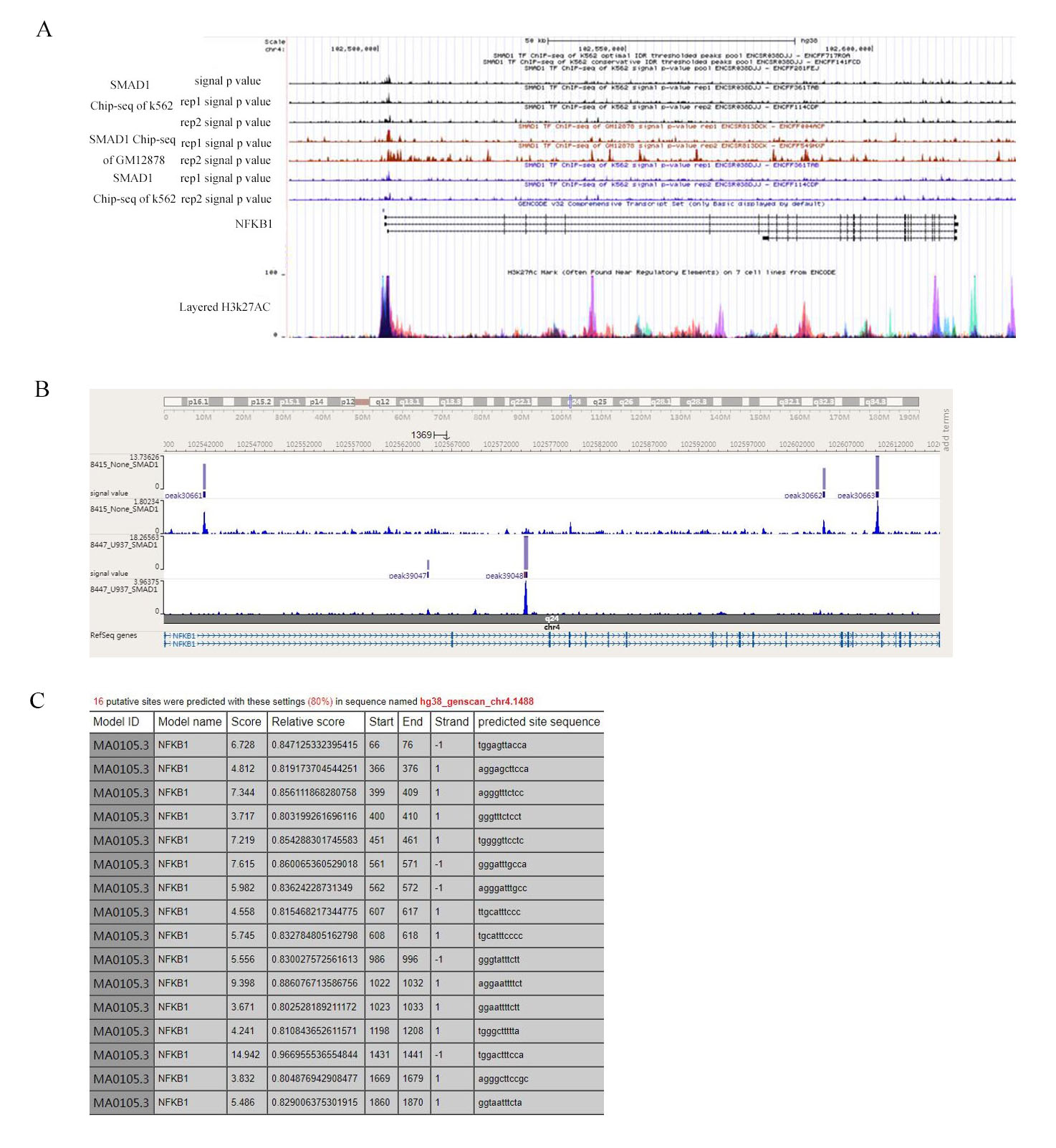


**Fig S5** (A)SMAD1 binding sties and histone modification around the NF-κB1 promoter identified by Chip-seq data from ENCODE. (B) Genome-wide identification of transcription binding sites of SMAD1 in the NF-κB1 promoter region based on UCSC data. (C) 16 putative sites were predicted with these setting (80%) in sequence named hg38_genscan_chr4.1488.


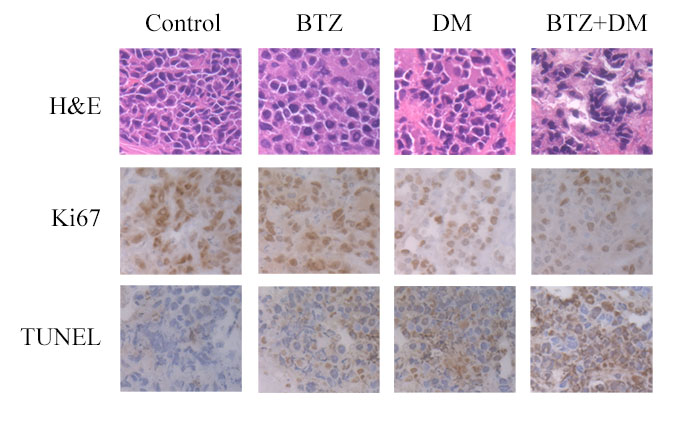


**Fig S6**. Representative microscopic images of tumor sections from four treated groups analyzed for histology (H&E), proliferation (Ki-67) or apoptosis (TUNEL).


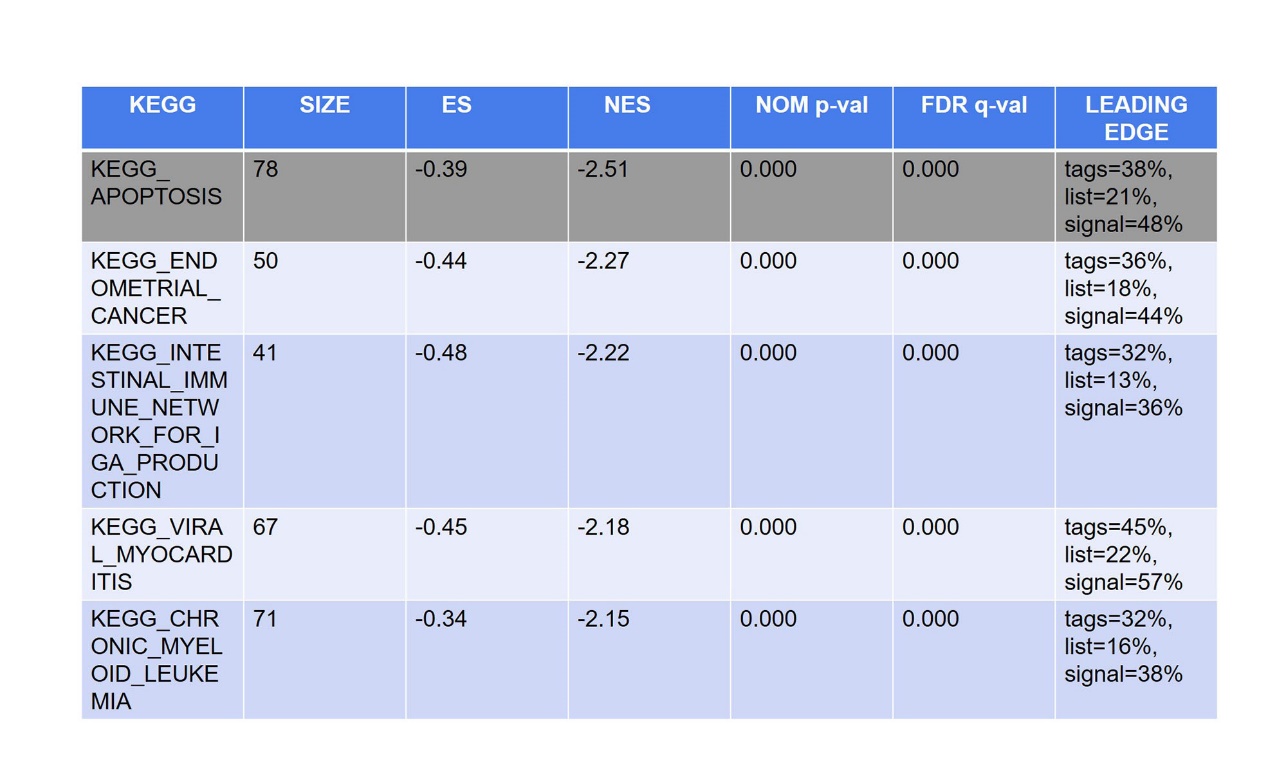


STable 1. The top 5 KEGG pathway enriched by GSEA in APEX trial dataset by comparing the SMAD1-high and SMAD1-low expression samples.


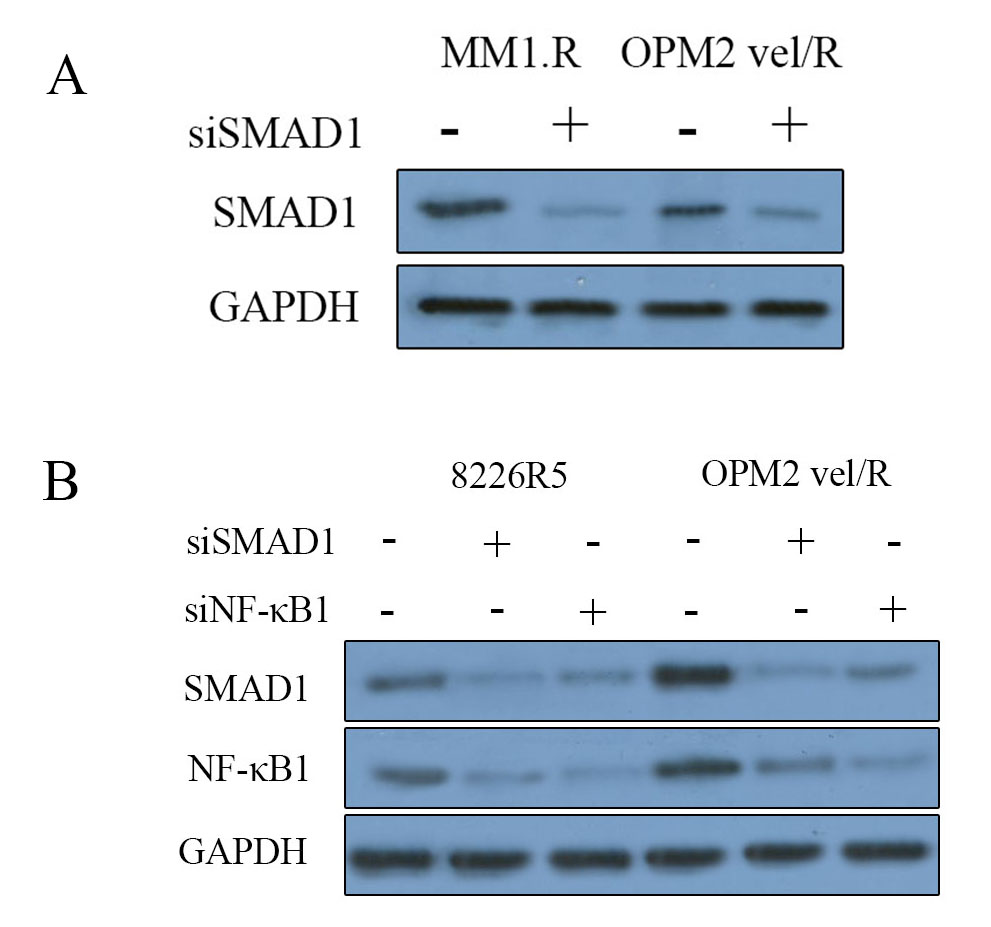


**Fig S7 (A)** In parallel with cell cycle assay, western blot was performed to measure the efficiency of siRNA in MM cell lines. (B) In parallel with immunofluorescence, Protein lysate was subjected to western blot with indicated antibodies.
